# Oligonucleotide Phosphorothioates Enter Cells by Thiol‐Mediated Uptake

**DOI:** 10.1002/anie.202107327

**Published:** 2021-07-21

**Authors:** Quentin Laurent, Rémi Martinent, Dimitri Moreau, Nicolas Winssinger, Naomi Sakai, Stefan Matile

**Affiliations:** ^1^ School of Chemistry and Biochemistry National Centre of Competence in Research (NCCR) Chemical Biology University of Geneva Geneva Switzerland

**Keywords:** cellular uptake, dynamic covalent chemistry, oligonucleotides, phosphorothioates

## Abstract

Oligonucleotide phosphorothioates (OPS) are DNA or RNA mimics where one phosphate oxygen is replaced by a sulfur atom. They have been shown to enter mammalian cells much more efficiently than non‐modified DNA. Thus, solving one of the key challenges with oligonucleotide technology, OPS became very useful in practice, with several FDA‐approved drugs on the market or in late clinical trials. However, the mechanism accounting for this facile cellular uptake is unknown. Here, we show that OPS enter cells by thiol‐mediated uptake. The transient adaptive network produced by dynamic covalent pseudo‐disulfide exchange is characterized in action. Inhibitors with nanomolar efficiency are provided, together with activators that reduce endosomal capture for efficient delivery of OPS into the cytosol, the site of action.

## Graphical abstract

Replacing one oxygen by one sulfur per monomer converts native oligonucleotides that cannot enter cells into cell‐penetrating oligophosphorothioates. The reason for this is thiol‐mediated uptake: dynamic covalent exchange with cellular thiols and disulfides that can be inhibited and activated according to the principles of dynamic covalent chemistry.

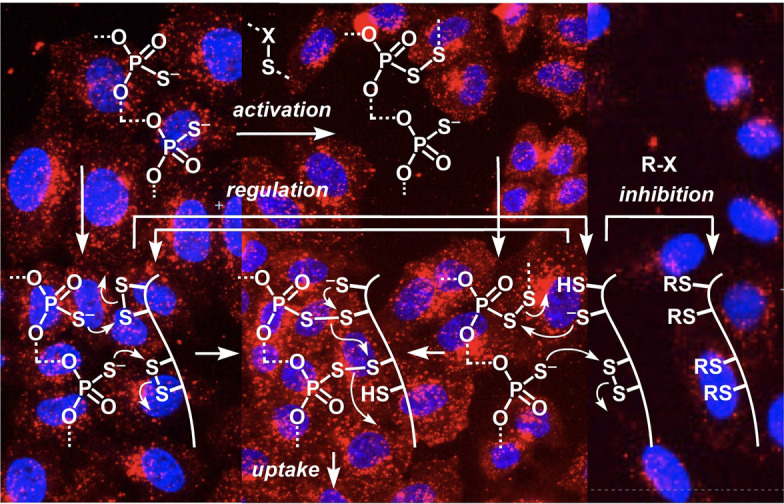

Of general significance for science and society in the broadest sense, oligonucleotide technology applications have often been hampered by poor cellular uptake.[^1^, ^2^, ^3^] Besides a large number of gene transfection vectors,[^1^, ^2^, ^3^] many non‐native modifications of oligonucleotides have been introduced in the past decades to address this challenge.^[4]^ Oligonucleotide phosphorothioates (OPS) such as **1**, where one oxygen atom of the bridging phosphodiesters of the biological original **2** is replaced by a sulfur atom, have been one of the first backbone modification introduced in the field (Figure 1).^[5]^ Apart from better nuclease stability and hydrophobicity, OPS have been shown to penetrate cells much more efficiently than unmodified DNA in the absence of transfecting agents (Figure 1 A, B).^[6]^ For these reasons, OPS have found applications in the past decades in the clinics, with several FDA‐approved drugs on the market and several in late clinical trials.^[7]^

**Figure 1:**
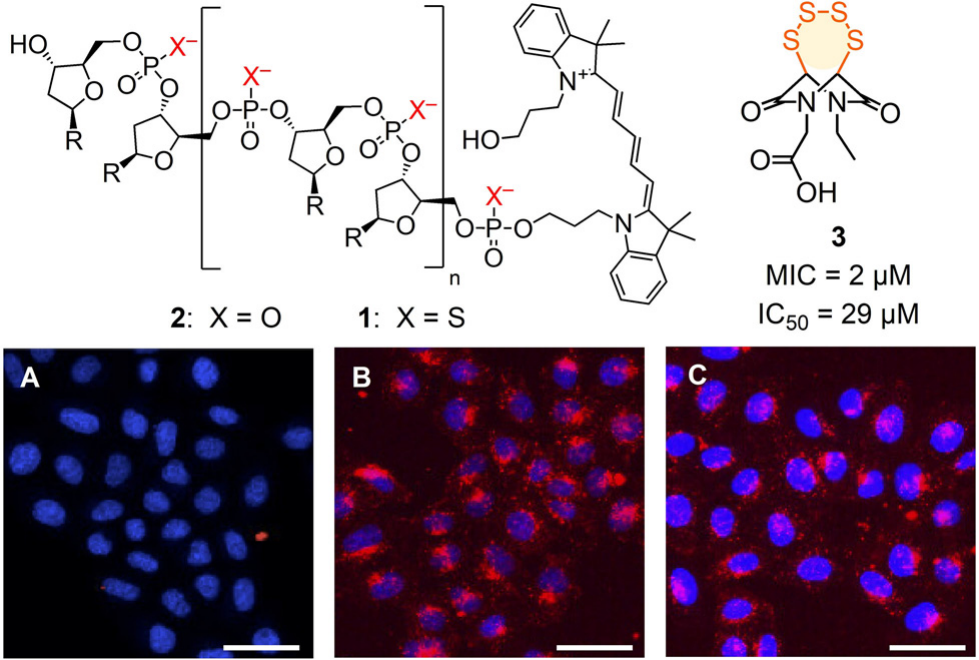
CLSM images of HeLa Kyoto cells after 2 h incubation with DNA **2** (A) and OPS **1** without (B) and with (C) preincubation with **3** (10 μM); red, **1**, **2** (Cy5); blue, Hoechst 33 342; scale bar: 50 μm. R=nucleobases, sequence: AGGTCCCCATACACCGAC.

Considering a p*K*_a_ close to zero for phosphorothioate monomers,^[8]^ transient protonation for transmembrane translocation in neutral form^[9]^ was not likely to account for cell penetration. Pioneering work by Crooke and co‐workers has shown that interactions with membrane‐bound proteins are essential for OPS uptake through endocytosis and other, unclear, pathways.[^7^, ^10^] Among identified protein partners, many of them are disulfide‐rich proteins.[^7^, ^10^, ^11^, ^12^, ^13^, ^14^, ^15^] This supported that dynamic covalent exchange chemistry[^1^, ^3^, ^16^, ^17^] might enable OPS to penetrate cells so easily. Thiol‐mediated uptake is emerging as method of choice to bring challenging substrates into cells and to hinder viral entry.[^1^, ^10^, ^17^, ^18^, ^19^] In the following, we report that thiol‐mediated uptake accounts for the entry of OPS, and show how this knowledge is of use to enhance uptake and reduce endosomal capture.

The occurrence of thiol‐mediated uptake is most convincingly demonstrated by the inhibition of the dynamic covalent exchange with the cell during uptake.^[10]^ Traditionally, this has been done with Ellman's reagent. Weak and unreliable, this single inhibitor has been replaced recently by a collection of inhibitors which are up to 5000 times more active and cover at least some of the different uptake pathways involved.^[20]^

From this collection, inhibitor candidates **3**–**12** were selected to explore interference with the uptake of OPS **1**, a random 18‐mer labelled with Cy5 at its 5′‐terminus (Figure 1, Figure 2; Table S1).^[20]^ If needed, inhibitors were prepared by multistep synthesis following reported procedures (see the Supporting Information). A recent automated high‐content high‐throughput (HCHT) screening assay^[20]^ was used to secure data from multiwell plates with thousands of HeLa Kyoto cells. They were first preincubated for 1 h with **3**–**12** at various concentrations below their toxicity limit^[20]^ and then, after inhibitor removal, incubated for 2 h with Cy5‐OPS **1**. The dependence of the uptake of **1** on the concentration of **3**–**12** (Figure 2 A) was then used to determine the MIC_OPS_ (Figure 2 B) and, if accessible, also the IC_50_, i.e., the concentration to observe ≈15 % and 50 % inhibition, respectively (Figures 1, 2, S10–S19, Table S2).

**Figure 2:**
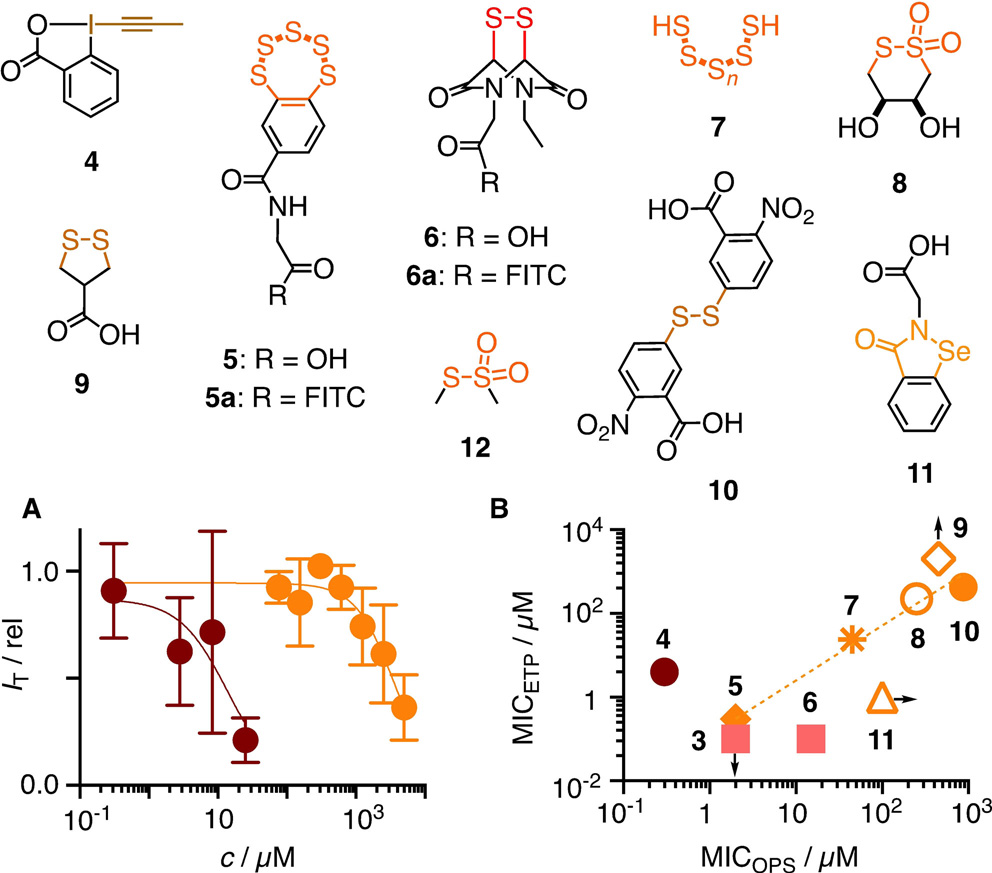
Inhibitors of the uptake of OPS **1**. A) Dose response curves for **4** (brown) and **10** (orange). *I*_T_: Average fluorescence intensities per cell ± SEM, normalized against that without the addition of an inhibitor (*I*_T(0)_=1), with fit to Hill equation. B) Comparison of MICs against OPS **1** and ETP **6 a** ^[20]^ (upward and rightward arrows: MIC > *c*_MAX_; downward arrow: MIC<*c*_MIN_), with trend line to guide the eye.

The best inhibitor of the uptake of OPS **1** was the hypervalent iodine reagent **4**, which irreversibly reacts with thiols on the cell surface.^[21]^ A sub‐micromolar MIC_OPS_=0.3 μM and an IC_50_=14 μM were obtained. Excellent inhibition of OPS uptake was also found for the most reactive cyclic oligochalcogenides (COCs), i.e., BPS pentasulfide **5**,^[22]^ ETP tetrasulfide **3** ^[22]^ and ETP disulfide **6**.^[23]^ The less reactive AspA[^10^, ^12^] **9** was also less impressive as inhibitor. The cyclic thiosulfonate COC **8** was of interest mostly because, among other activities,^[14]^ it best inhibited the cellular entry of SARS‐CoV‐2 virus models.^[20]^ In agreement with the importance of adaptive networks from exchange cascades, inorganic polysulfide **7** was recorded as good inhibitor, while ebselen analog **11** as well as MMTS **12** were inactive. The Ellman control **10** was as poor as expected.^[20]^

Comparison with results from the inhibition of the thiol‐mediated uptake of fluorescent ETP **6 a** revealed an overall positive correlation of MIC_OPS_ and MIC_ETP_, consistent with their reactivities, but with some distinct outliers (Figure 2 B). This mismatch has been observed previously comparing **6 a** and **5 a** and interpreted as support for the existence of multiple targets.^[20]^ Multiple pathways available for thiol‐mediated uptake were implied also from proteomics analysis, which revealed, inter alia, that the entry of AspA **9** is dependent on the transferrin receptor, while ETP **6** is not.[^12^, ^23^] Compared to ETP **6 a**, uptake inhibition of OPS **1** was better with irreversible **4** and AspA **9** but weaker with ETP **6**. This could support pathways including the transferrin receptor, which would agree with significant endosomal capture (vide infra).^[10]^ High activity of ETP **3** was intriguing because, contrary to the contracted **6**, the expanded **3** is a poor transporter.^[22]^ Ebselen analog **11**, an inhibitor of the entry of **6 a** (unpublished) and SARS‐CoV‐2,^[24]^ was completely inactive up to its solubility limit. In contrast, the anomalous dose response of Ellman's reagent **10** against **6 a** ^[20]^ converted into normal curves with OPS **1**, which provided also access to an IC_50_=3.6 mM.

The overall positive correlation in inhibition patterns of OPS and ETP **6 a** uptake (Figure 2 B) might imply that the many proteins known from OPS uptake^[7]^ contribute to the entry of COCs and viruses. SCARB1, for example, is involved in the uptake of OPS,^[7]^ SARS‐CoV‐2,^[11]^ hepatitis virus,^[10]^ and COC **9**.^[12]^ Hinting toward thiol‐mediated uptake as a unifying network coding for entry, similar, at least partial coincidences can be found for other target candidates (e.g. the transferrin receptor,[^10^, ^12^] EGFR,[^7^, ^10^, ^14^] integrins,[^7^, ^25^] CLIC[^7^, ^12^]).

Thiol‐mediated uptake is defined as enhanced, inhibitable uptake in the presence of dynamic covalent chalcogen exchangers, usually disulfides.^[10]^ This cascade exchange chemistry occurs during uptake via direct translocation, fusion or endocytosis, involves multiple protein targets (vide supra) and, presumably, also transient micellar membrane microdomains, at least for the cytosolic delivery of large substrates.^[10]^ In this context, the inhibition of OPS uptake by thiol‐reactive probes was intriguing because the multivalent OPS **1** would be expected to exchange with disulfides rather than with thiols on the cell surface (Figure 3 A, **II**) to trigger the exchange cascades expected for efficient uptake (**III**). Surprisingly little is known about the dynamic covalent exchange chemistry of phosphorothioates.[^26^, ^27^] Thus, 5′‐AMPS **13** was used as minimalist OPS to explore the possible exchange with the non‐activated disulfides **14** of cystine residues on cell surfaces. The in situ formation of pseudo‐disulfides **15** and their reduction with TCEP could be demonstrated by HPLC and MS (Figures 3 B, S31). In contrast, 5′‐AMPS **13** did not react with reduced cysteines (Figure S32).

**Figure 3:**
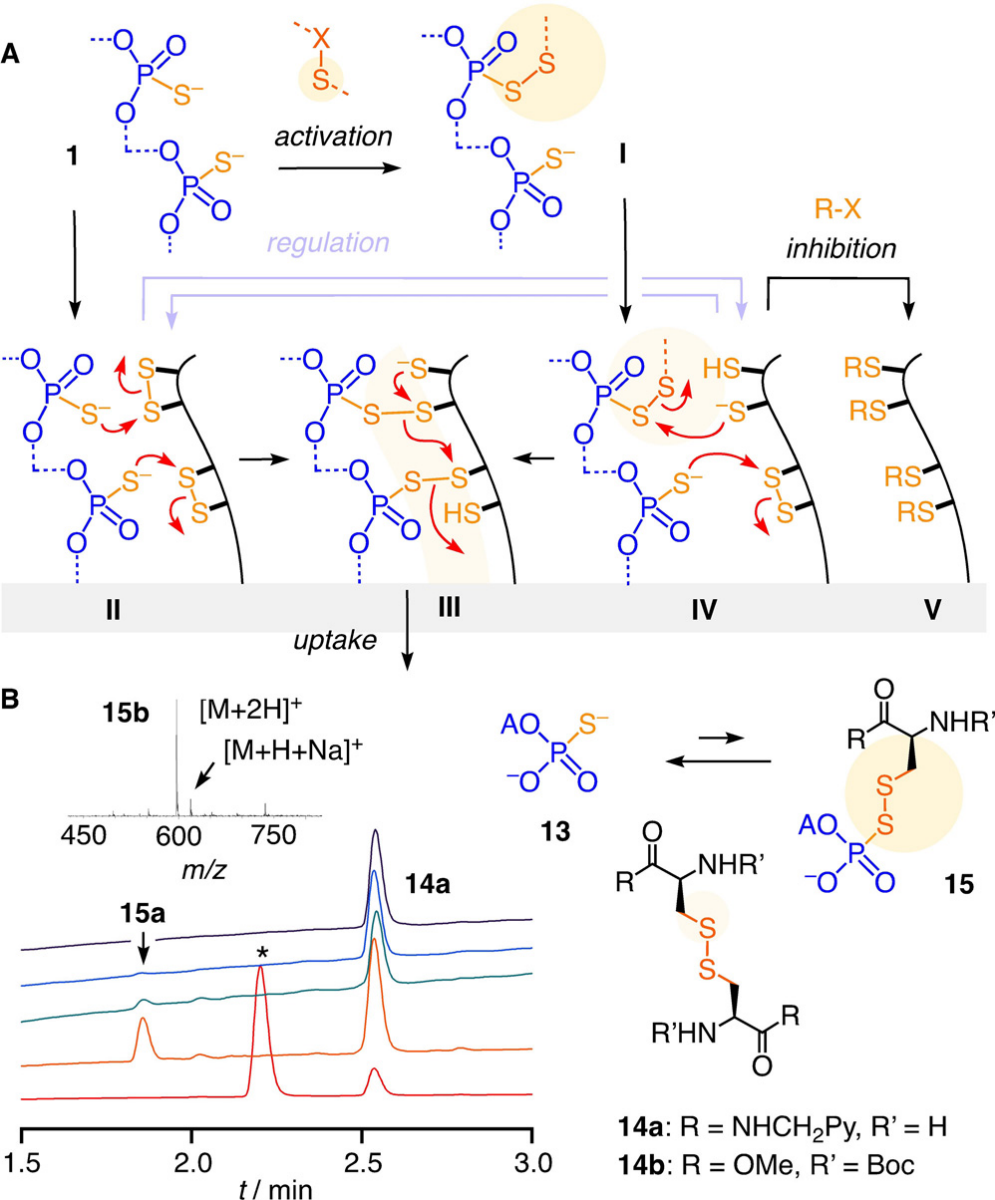
A) A tentative dynamic covalent exchange mechanism for the thiol‐mediated uptake of OPS. B) Normalized HPLC traces for the exchange of **13** (A=adenosyl) with **14 a** (top to bottom: **14 a**, 1:1, 10:1, 100:1 **13**/**14 a** and 100:1 after addition of 100 equiv. of TCEP), and mass spectrum of **15 b**. Conditions: 100 μM **14 a**, PBS, pH 7.4; *=reduced **14 a**.

These results supported that the dynamic covalent chemistry of OPS **1** operates with non‐activated disulfides but not with thiols (Figure 3, **II**). The efficient inhibition with thiol‐reactive agents thus implied that the blocking of exofacial thiols also led to the blocking of exofacial disulfides via biological regulation by, e.g., PDI (protein disulfide isomerase) or glutathione, to ultimately inactivate all accessible sulfur, thiols and disulfides (**V**).[^10^, ^18^, ^28^] Alternatively, or in addition, our inhibition results could imply that OPS **1** is dynamic covalently activated by extracellular disulfides near the cell surface, e.g., oxidized glutathione, and the resulting, transient pseudo‐disulfides **I** then exchange with exofacial thiols (**IV**) to end up with the same exchange cascade (**III**).

The possibility to activate OPS **1** in situ as pseudo‐oligochalcogenides **I** was intriguing (Figure 3 A). The same inhibitors identified above could conceivably act as activators, depending on conditions. While their incubation with cells removes cell surface thiols and thus inhibits thiol‐mediated uptake (Figure 3 A, **V**), the complementary incubation with OPS **1** could possibly afford pseudo‐oligochalcogenides to facilitate exchange with cell surface thiols and thus activate thiol‐mediated uptake (Figure 3 A, **I**). The exchange of 5′‐AMPS **13** with DTNB **10** to produce activated pseudo‐disulfides was confirmed^[26]^ easily (Figure S29). MMTS **12** was found to exchange most efficiently with **13**, with only 4 equivalents needed to reach full conversion into pseudo‐disulfide **16** (Figures 4 G, S30). Exchange of phosphorothioate monomer **17** with BPS **5 a** triggered the emergence of an adaptive dynamic polysulfide network^[22]^ on the OPS model **18** (Figure 4 H). With the same HPLC‐MS fingerprinting, transient, dynamic covalent exchange activation was confirmed also for activators **3** and **7** (Figures S27, S28).

**Figure 4:**
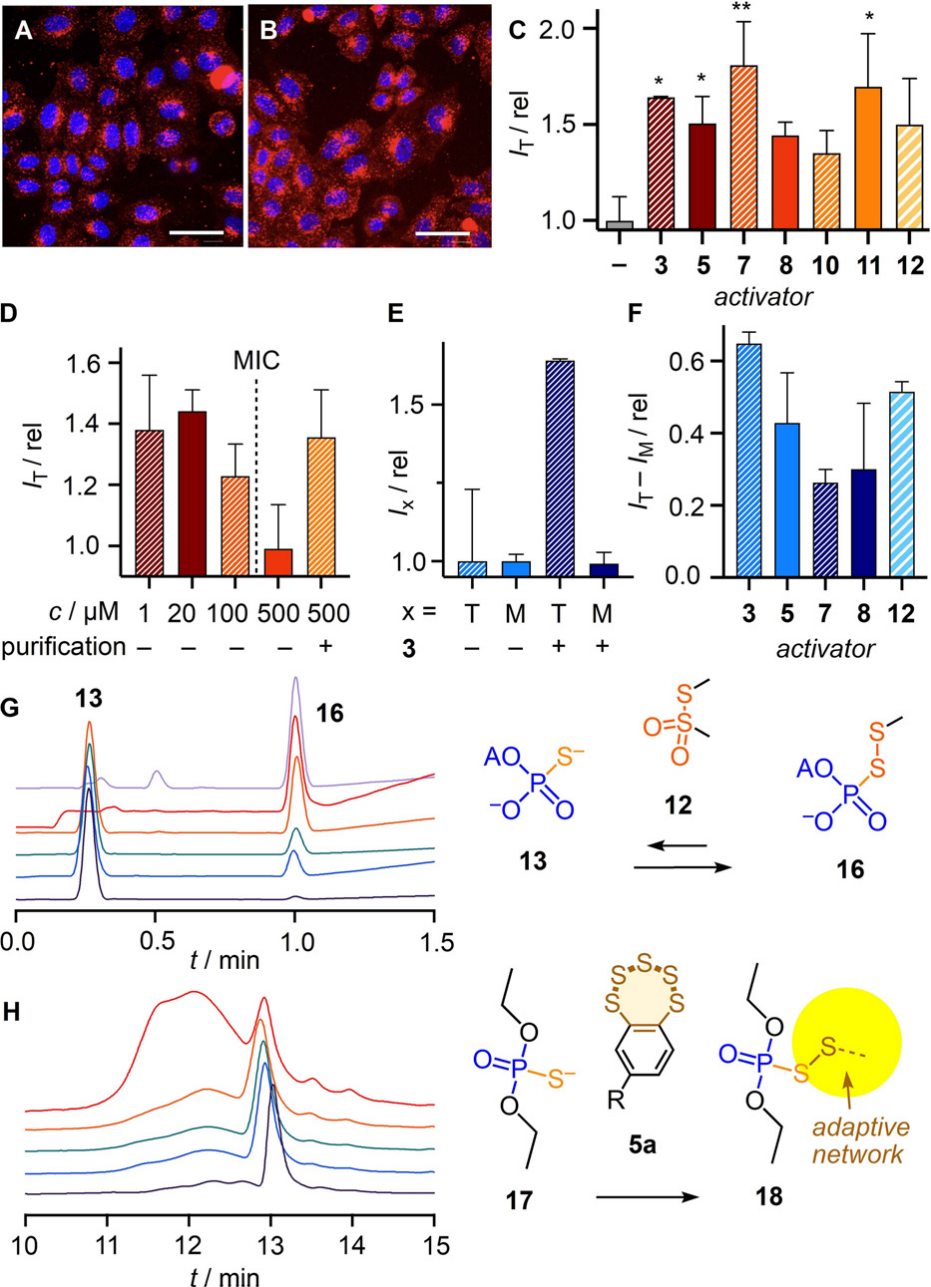
CLSM images of HeLa Kyoto cells after 2 h incubation at 37 °C with A) non‐modified OPS **1** and B) activated OPS **I** (500 nM **1**, 500 μM **3**, PBS, pH 7.4, 30 min, 25 °C, followed by centrifugal filtration), scale bar=50 μm. C) HCHT data showing normalized *I*_T_, i.e., fluorescence intensity per cell *I* of the whole cell (T) for OPS **1** activated with **3** (500 μM, with purification after activation), **5** (500 μM, with purification), **7** (25 μM), **8** (20 μM), **10** (500 μM), **11** (50 μM) and **12** (50 μM), divided by fluorescence intensity per cell *I*_0_ of non‐activated OPS **1**. Data are average values from > two sets of experiments ± SEM and analyzed by one‐way ANOVA compared to that without activation (* *P*<0.033; ** *P*<0.0021). D) *I*_T_ for OPS **1** (500 nM) activated with **8** (*c* varied) with and without purification after activation. E) Normalized fluorescence intensities in whole cells (*I*_T_) vs. those in punctate emission (*I*_M_) of OPS **I** activated with **3** (dark blue) relative to nonactivated OPS **1** (light blue). F) *I*_T_−*I*_M_ for OPS **1** activated with **3**, **5**, **7**, **8**, and **12**. G) Normalized HPLCs of **13** with 0.1, 0.2, 0.5, 1.0, 4.0 and 10 equiv. **12** (bottom to top). H) Same for **5 a** with 0.0, 0.5, 1.0, 10 and 100 equiv. **17**.

To activate in situ for thiol‐mediated uptake, OPS **1** was incubated for 30 min with various concentrations of potential activators (Figures S20–S26). Added as mixtures, the uptake of OPS increased with increasing concentrations of activators up to concentrations close to the MIC, beyond which the leftover activator started to inhibit uptake (e.g., **8**, Figures 4 D, S20, S21, S23). The removal of the excess activator was possible but not always beneficial, since this led to a partial loss of activation due to the transient nature of activated OPS **I**, as confirmed by HPLC (e.g., Figure S28). Thus, transient activation of OPS **1** was examined without purification, which limited activation to concentrations below the onset of overcompeting inhibition (Figure 4 D). With ETP **3**, BPS **5**, polysulfides **7**, and thiosulfonates **8** and **12**, activation remained partially preserved after removal of excess activator, resulting in up to ≈1.7‐fold increase with **3** (Figures 4 A–C, S20).

Like the transferrin‐receptor dependent AspA **9**, OPS are known to enter cells mostly by endocytosis, localizing in early and late endosomes within 10–50 min and in lysosomes afterwards, with activities observed only after several hours, indicating that endosomal escape is slow.^[6]^ After OPS activation, more diffuse fluorescence was observed in CLSM images (Figure 4 A, B). Image analysis was performed to segment punctate structures that, with all likelihood, correspond to endolysosomes. Whereas the integrated intensity in the whole cell increased upon activation, the integrated fluorescence intensity remained constant in the mask (Figure 4 E). This difference indicated that the increase of uptake is correlated with a more diffuse, most likely cytosolic localization. The same shift from endosomal to cytosolic location was observed for all tested activators (Figure 4 F). Activation of OPS uptake by in situ formation of pseudo‐oligosulfides **I** presumably induces a shift of reactivity to include different target proteins, seemingly leading to either direct translocation or facilitated endosomal escape (Figure 3 A). To close, we reiterate that HCHT imaging automatically informs on cell viability, and that all reported data were obtained at concentrations below the onset of toxicity (which already has been reported for most inhibitors used^[20]^).

In summary, the biology of OPS is understood and not topic of this study. It is also known that the success of OPS in biology and medicine originates in part from their ability to penetrate cells, and several proteins have been identified to contribute to endocytosis and other, unknown mechanisms. What has remained mysterious is the question why the replacement of one oxygen by one sulfur per monomer in the backbone converts an oligonucleotide that cannot penetrate cells into one that can. Here we show that the underrecognized dynamic covalent exchange of phosphorothioates with cellular thiols and disulfides accounts for the cell penetration, and that their thiol‐mediated uptake can be inhibited and activated according to the general principles of dynamic covalent sulfur exchange chemistry, from simple pseudo‐disulfides over thiosulfonates to more complex adaptive networks.

## Conflict of interest

The authors declare no conflict of interest.

## Supporting information

As a service to our authors and readers, this journal provides supporting information supplied by the authors. Such materials are peer reviewed and may be re‐organized for online delivery, but are not copy‐edited or typeset. Technical support issues arising from supporting information (other than missing files) should be addressed to the authors.

Supporting Information
