# Supplementary material for: Oligonucleotide Phosphorothioates Enter Cells by Thiol‐Mediated Uptake
Source: Angew Chem Int Ed Engl. 2021 Jul 21;60(35):19102–6. doi: 10.1002/anie.202107327 (PMC8456962; doi:10.1002/anie.202107327)
Supplement: Supplementary file 1 — Supporting Information [file ANIE-60-19102-s001.pdf]

Angewandte Chemie
Eine Zeitschrift der Gesellschaft Deutscher Chemiker

Supporting Information

Oligonucleotide Phosphorothioates Enter Cells by Thiol-Mediated Uptake

*Quentin Laurent, Rémi Martinent, Dimitri Moreau, Nicolas Winssinger, Naomi Sakai, and Stefan Matile**

anie_202107327_sm_miscellaneous_information.pdf

Table of Content

1. Materials and Methods S3

2. Synthesis S5

 2.1. Synthesis of **14a** S5

 2.2. Synthesis of Inhibitors S6

 2.3. Sequences of OPS and DNA Strands S6

3. Cell Culture S6

4. High-Content High-Throughput (HCHT) Inhibitor Screening S7

 4.1. General Procedure for HCHT Inhibitor Screening S7

 4.2. Data Analysis for HCHT Inhibitor Screening S8

 4.3. Optimization of Uptake Conditions S13

 4.4. Inhibitor Screening S15

5. Activation of OPS Uptake S20

 5.1. General Procedure for OPS Activation S20

 5.2. HCHT Data of OPS Activation S21

6. HPLC Studies S24

 6.1. General Procedure for HPLC Studies S24

 6.2. Activation of BPS Library S25

 6.3. Di- and Polysulfide Exchange with 5'-AMPS S25

7. NMR Spectra S32

8. Supporting References S33

1. Materials and Methods

As in ref. S1. Briefly, reagents for synthesis were purchased from Fluka, Sigma-Aldrich, TCI, and Acros. Salts of the best grade available from Fluka or Sigma-Aldrich were used as received. Custom OPS and DNA were purchased from Sigma-Aldrich and used as received. Phosphate buffered saline (PBS, pH = 7.4), DMEM (GlutaMAX, 4.5 g/L D-glucose, with phenol red) medium, FluoroBrite DMEM (high D-Glucose) medium, Penicillin- Streptomycin, Fetal Bovine Serum, TrypLE Express Enzyme and V96-MicroWell platen were obtained from Thermo Fisher Scientific. µ-Plate 96-Well Black were obtained from Ibidi. Hoechst 33342 (10 mg/mL solution in water) was obtained from Invitrogen by Thermo Fisher Scientific. Analytical thin layer chromatography (TLC) were performed on silica gel 60 (Merck, 0.2 mm). Reverse phase flash chromatography was performed on a Biotage® Isolera Spektra. LCMS were recorded using a Thermo Scientific Accela HPLC equipped with a Thermo C_{18} Hypersil GOLD column (50 x 2.1 mm, 1.9 µm particles size) coupled with a LCQ Fleet three-dimensional ion trap mass spectrometer (ESI, Thermo Scientific) with a linear elution gradient from 95% H_2O / 5% CH_3CN + 0.1% TFA to 10% H_2O / 90% CH_3CN + 0.1% TFA in 4.0 minutes at a flow rate of 0.75 mL/min. IR spectra were recorded on a Perkin Elmer Spectrum 100 FT-IR spectrometer (ATR, Golden Gate) and are reported as wavenumbers υ in cm^{-1} with band intensities indicated as s (strong), m (medium), w (weak), br (broad). All ^{1}H and ^{13}C NMR spectra were recorded (as indicated) on a Bruker 300 MHz, 400 MHz or 500 MHz spectrometer at room temperature (25 °C) and are reported as chemical shifts (δ) in ppm relative to TMS ($\delta = 0$). Spin multiplicities are reported as a singlet (s), doublet (d), triplet (t), quartet (q), and quintet (p) with coupling constants (J) given in Hz, or multiplet (m). Broad peaks are marked as br. ^{1}H and ^{13}C resonances were assigned with additional information from 1D and 2D NMR spectra (H,H-COSY, DEPT 135, HSQC and HMBC). ESI-HRMS was measured on Xevo G2-S Tof (Waters). All mass data are reported as mass-per-charge ratio m/z (intensity in %, [assignment]).

Abbreviations. 5'-AMPS: Adenosine-5'-O-monophosphorothioate; BPS: Benzopolysulfane; CLSM: Confocal Laser Scanning Microscopy; CV: Column volume; DMEM: Dulbecco's Modified Eagle Medium; DMF: Dimethylformamide; DMSO: Dimethylsulfoxide; DTNB: 5,5'-dithiobis-(2-nitrobenzoic acid); ESI: Electrospray ionization; ETP: Epidithiodiketopiperazine; FBS: Fetal bovine serum; HBTU: 2-(1H-Benzotriazole-1-yl)-1,1,3,3-tetramethyluronium hexafluorophosphate; HCHT: High-content high-throughput; HPLC: High pressure liquid chromatography; HRMS: High-resolution mass spectroscopy; IPS: Inorganic polysulfide; MIC: Minimum inhibitory concentration; MMTS: S-methyl methanethiosulfonate; OPS: Oligonucleotide phosphorothioate; PBS: Phosphate buffer saline; PFA: Paraformaldehyde; rt: Room temperature; TEAA: Tetraethylammonium acetate; TFA: Trifluoroacetic acid.

2. Synthesis

2.1. Synthesis of 14a

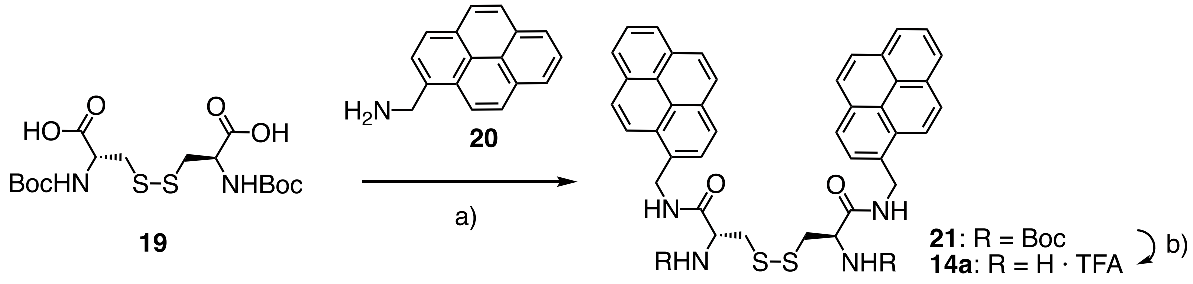

Scheme S1. (a) HBTU, Et_3N, DMF, rt, 20 min. (b) TFA, CH_2Cl_2, rt, 20 min, 2 steps, 72%.

Compound 21. To a solution of Boc-Cystine (**19**, 30 mg, 68 μmol) in DMF (0.7 mL) and CH_2Cl_2 (0.7 mL) were added HBTU (62 mg, 0.16 mmol), 2,4,6-collidine (22 μL, 0.17 mmol), **20** (HCl salt, 37 mg, 0.14 mmol) and Et_3N (20 μL, 0.14 mmol). The mixture was stirred for 20 min at rt and CH_2Cl_2 was evaporated under reduced pressure. The resulting suspension was diluted with H_2O (≈5 mL), sonicated, centrifuged, and the supernatant was discarded. The residue was suspended in MeOH (≈5 mL), sonicated, centrifuged, and the supernatant was discarded. The same procedure was repeated two more times. The poorly soluble product **21** (50 mg) was used in the next reaction without further purifications.

Compound 14a. To a suspension of **21** (50 mg) in CH_2Cl_2 (2 mL) were added *i*Pr_3SiH (80 μL, 0.26 mmol) and TFA (1 mL). The resulting solution was stirred at rt for 20 min, and then concentrated to dryness. The residue was triturated with Et_2O and purified by reverse phase flash chromatography (Claricep C18 12 g, CH_3CN + 0.1% TFA / H_2O + 0.1% TFA 2:8 for 1 CV, linear gradient 2:8 to 8:2 in 10 CV, elution at 6.8 CV) to give pure **14a** (42 mg, 2 steps 72%) as a colorless solid. $[\alpha]_D^{20}$ -43 (*c* 0.79, MeOH); IR (neat): 3302 (w), 3041 (w), 1666 (s), 1554 (m), 1431 (w), 1204 (s), 1137 (m), 841 (s), 8001 (w), 757 (w), 724 (w), 708 (w), 681 (w); ^{1}H NMR (400 MHz, CD_3OD): 8.02 (dd, $^3J_{H\text{-}H}$ = 7.7 Hz, $^4J_{H\text{-}H}$ = 1.1 Hz, 2H), 7.96 – 7.88 (m, 6H), 7.86 (d, $^3J_{H\text{-}H}$ = 9.4 Hz, 2H), 7.83 (t, $^3J_{H\text{-}H}$ = 7.9 Hz, 2H), 7.77 (s, 4H), 7.68 (d, $^3J_{H\text{-}H}$ = 7.8 Hz, 2H), 4.81 (d, $^2J_{H\text{-}H}$ = 14.4 Hz, 2H), 4.54

(d, $^2J_{H-H}$ = 14.4 Hz, 2H), 4.21 (dd, $^3J_{H-H}$ = 7.7, 4.7 Hz, 2H), 3.31 – 3.26 (m, 2H), 3.02 (dd, $^2J_{H-H}$ = 14.8 Hz, $^3J_{H-H}$ = 7.7 Hz, 2H); ^{13}C NMR (101 MHz, CD_3OD): 168.5 (C), 132.6 (C), 132.4 (C), 131.8 (C), 131.1 (C), 130.0 (C), 129.1 (CH), 128.5 (CH), 128.4 (CH), 128.1 (CH), 127.0 (CH), 126.4 (CH), 126.3 (CH), 125.8 (C), 125.7 (CH), 125.6 (C), 123.4 (CH), 52.9 (CH), 42.9 (CH_2), 41.3 (CH_2); HRMS (ESI, +ve) calcd for $C_{40}H_{34}N_4O_2S_2$ $[M+H]^+$: 667.2197, found: 667.2214.

2.2. Synthesis of Inhibitors

Compounds 3, **5**, **6**, **7**, **8** and **9** were synthesized and purified according to procedures described in ref. S1.

Compound 4 was synthesized according to a procedure described in ref. S2.

Compound 11 was synthesized according to a procedure described in ref. S3.

2.3. Sequences of OPS and DNA Strands

Table S1. Sequences of OPS and non-modified DNA strands used in this study

Compound	Sequence
**1**[a]	Cy5-*A*G*G*T*C*C*C*C*A*T*A*C*A*C*C*G*A*C
**2**[a]	Cy5-AGGTCCCCATACACCGAC

[a]Random 18-mer. * indicates of phosphorothioate linkage.

3. Cell Culture

Human cervical cancer-derived HeLa Kyoto cells were cultured in DMEM (GlutaMAX, 4.5 g/L D-glucose, with phenol red) medium containing 10% fetal bovine serum (FBS) and 1% Penicillin/Streptomycin (PS). The cells were grown at 37 °C under 5% CO_2 on a 25 cm^3 tissue culture flask (TPD Corporation). Cells were detached by treatment with 1.5 mL of TrypLE Express at 37 °C for 5 min, followed by the addition of 6 mL of DMEM (GlutaMAX, 4.5 g/L D-glucose, with phenol

red) medium at 37 °C. The cells were resuspended in DMEM (GlutaMAX, 4.5 g/L D-glucose, with phenol red) medium and plated according to the concentration needed.

4. High-Content High-Throughput (HCHT) Inhibitor Screening

4.1. General Procedure for HCHT Inhibitor Screening

HeLa Kyoto cells were seeded at 8 $\times 10^4$ cells/well in FluoroBrite DMEM + 10% FBS on µ-Plate 96-well Black ibiTreat sterile and kept at 37 °C with 5% CO_2 overnight. Next day, serial dilutions of the inhibitors in PBS (10x final concentration), OPS (10x in PBS) and a solution of Hoechst 33342 (100 µg/mL) in PBS were prepared freshly in a 96-well V-bottom plate. Then, cells were washed with PBS (3 x 3 mL/well) and the media was exchanged to FluoroBrite DMEM (4 x 150 µL/well) using a plate washer (Biotek EL406®), keeping a final volume of 135 µL/well. The inhibitor solutions from the V-bottom plate were added to the cells (15 µL/well, 10x final concentration in PBS) using an electronic multichannel pipette to reach a final volume of 150 µL/well. Cells were incubated for 1 h at 37 °C with 5% CO_2. After this, cells were washed again using the plate washer and OPS solution (10 x in PBS) from the V-bottom plate was added (15 µL/well) using an electronic multichannel pipette to reach a final volume of 150 µL/well. After 2 h of incubation at 37 °C with 5% CO_2, the solution of Hoechst 33342 from V-bottom plate was added (15 µL/well) using an electronic multichannel pipette. After 15 min of incubation at 37 °C with 5% CO_2, the liquid was removed and cells were fixed with 3% PFA (80 µL/well). The plate was washed with PBS (9 x 3 mL/well) and imaged using an automated confocal microscope.

For each experiment, 6 images at 20x were recorded per well using two channels: blue for Hoechst 33342 (excitation filter: 377/50 nm; emission filter: 477/60 nm) and red for Cy5 (excitation filter: 620/50 nm; emission filter: 690/50 nm), as shown in Figure S1-S2. Duplicates were performed for each condition.

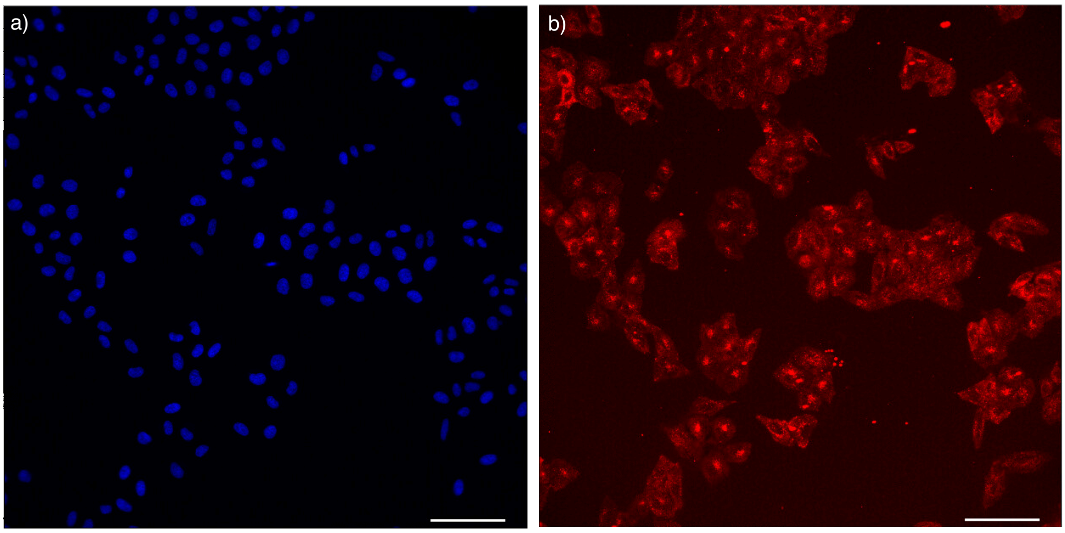

Figure S1. CLSM images of a) blue channel recording the Hoechst 33342 localization in the nuclei and b) red channel recording the signal from Cy5-labelled OPS **1**. Scale bar = 100 µm.

4.2. Data Analysis for HCHT Inhibitor Screening

For each cell, the blue channel image is used for the segmentation of nuclei and whole cell body.

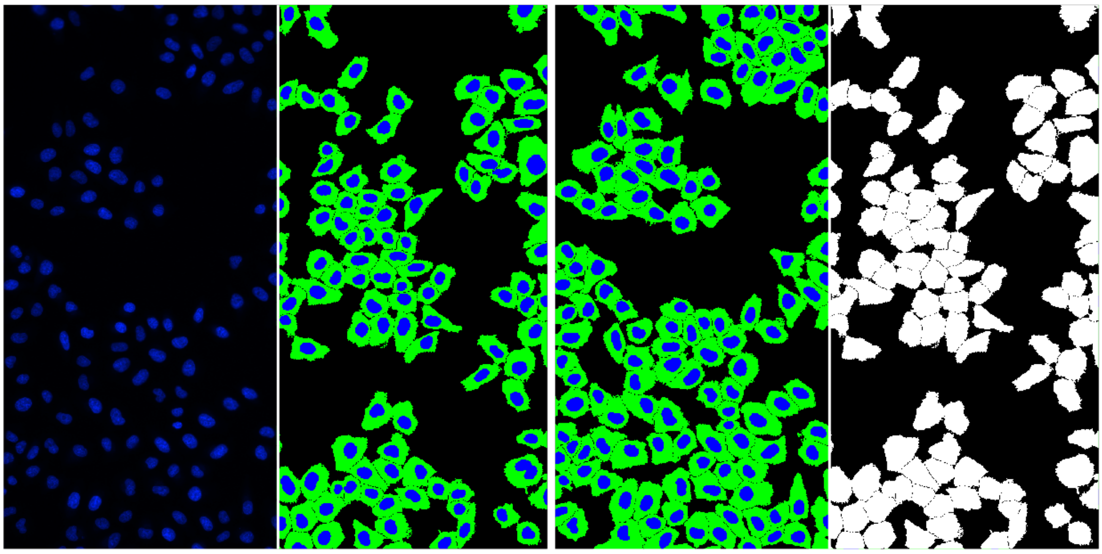

Figure S2. Segmentation of nuclei (blue mask) and cell body (green mask) using the blue channel.

Bright fluorescent aggregates were detected using a detection threshold of 5000 over the background intensity and grown to be removed.

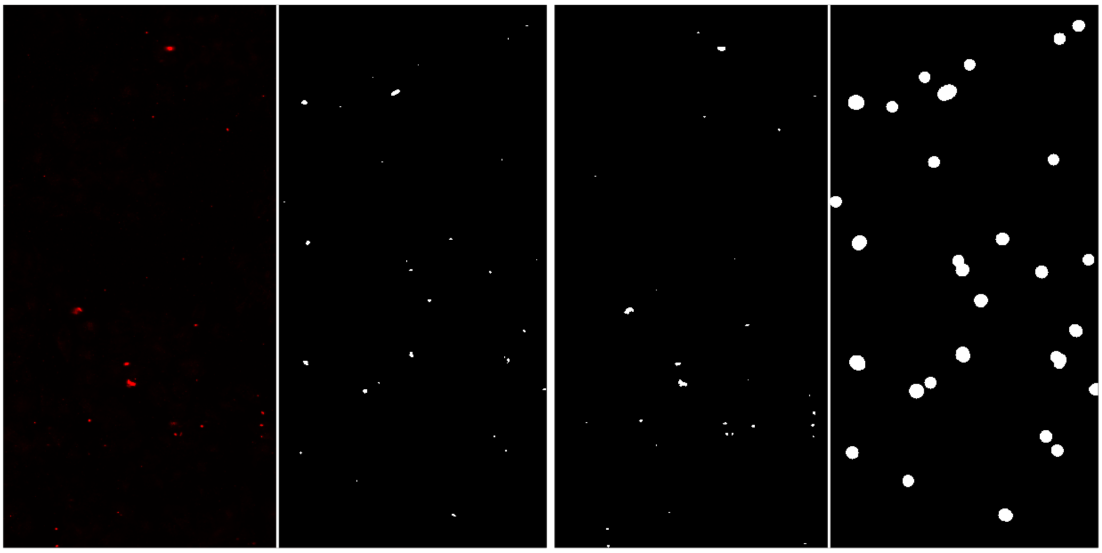

Figure S3. Detection of bright fluorescent aggregates using the red channel (left) and growth of the detected objects to be removed (right).

Masks of bright fluorescent aggregates were used as a proxy to remove all cells touched by them from the final quantification.

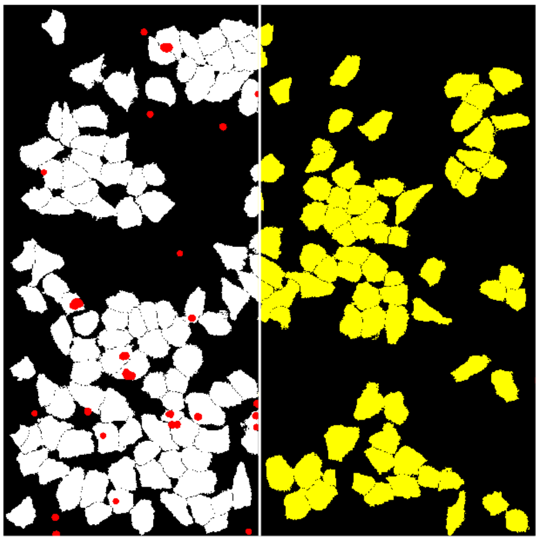

Figure S4. Detection of bright fluorescent aggregates using the red channel (left) and growth of the detected objects to be removed (right).

Cell body and dots masks were applied to extract the integrated (sum of the intensities of the pixels included in the mask) and average intensity values in the background.

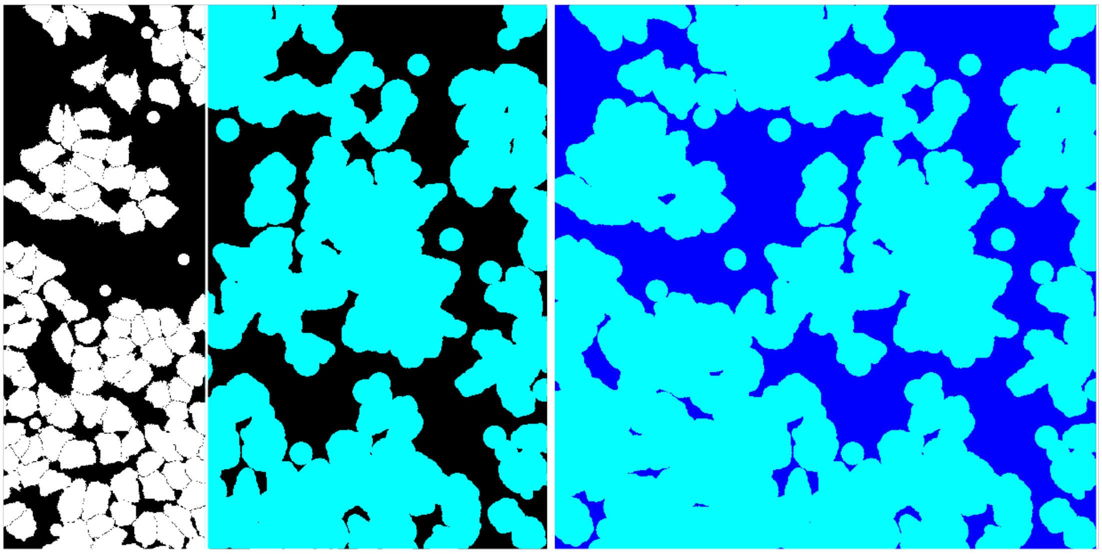

Figure S5. Mask (sky blue) applied for the quantification of the fluorescence intensity of background (dark blue) in the red channel image.

Integrated and average intensity of the detected and validated cells (~800 cells/well) was finally extracted from the mask.

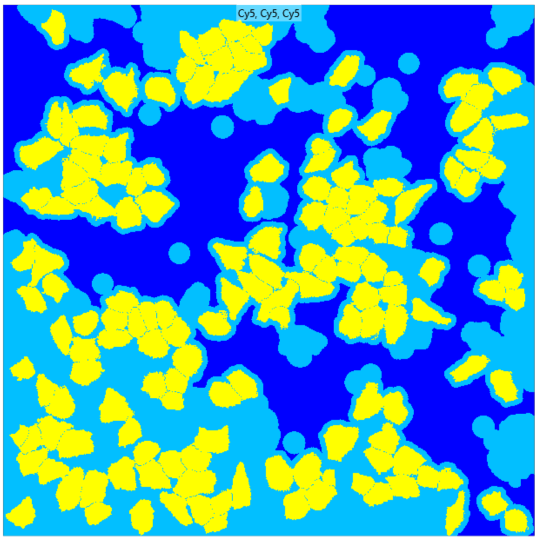

Figure S6. Analysis of the red channel image showing detected cells (yellow), excluded areas (sky blue) and background (dark blue).

Average fluorescent intensity values per cell (I) were calculated by subtracting average background intensity from average intensity of the whole cell. Average intensities I for each condition in the presence of inhibitors were normalized against that obtained without the addition of inhibitors (I_0) using Equation (S1).

$$I_T = I / I_0 \tag{S1}$$

Duplicates were performed for each condition and averaged. The resulting dependence of the relative fluorescent intensity values (I_T) to the concentration of inhibitors ($c_{inhibitor}$) was plotted and fitted with Equation (S2) to retrieve the half maximal inhibitory concentration (IC_{50}) and the Hill coefficient (n). MIC values were estimated from the fit curve as the concentration at which 15% of uptake was inhibited.

$$I_{rel} = 1 / (1 + (IC_{50} / c_{inhibitor})^{-n}) \tag{S2}$$

Top-hat transformation of the red channel image is used to facilitate the segmentation of the dotted structures.

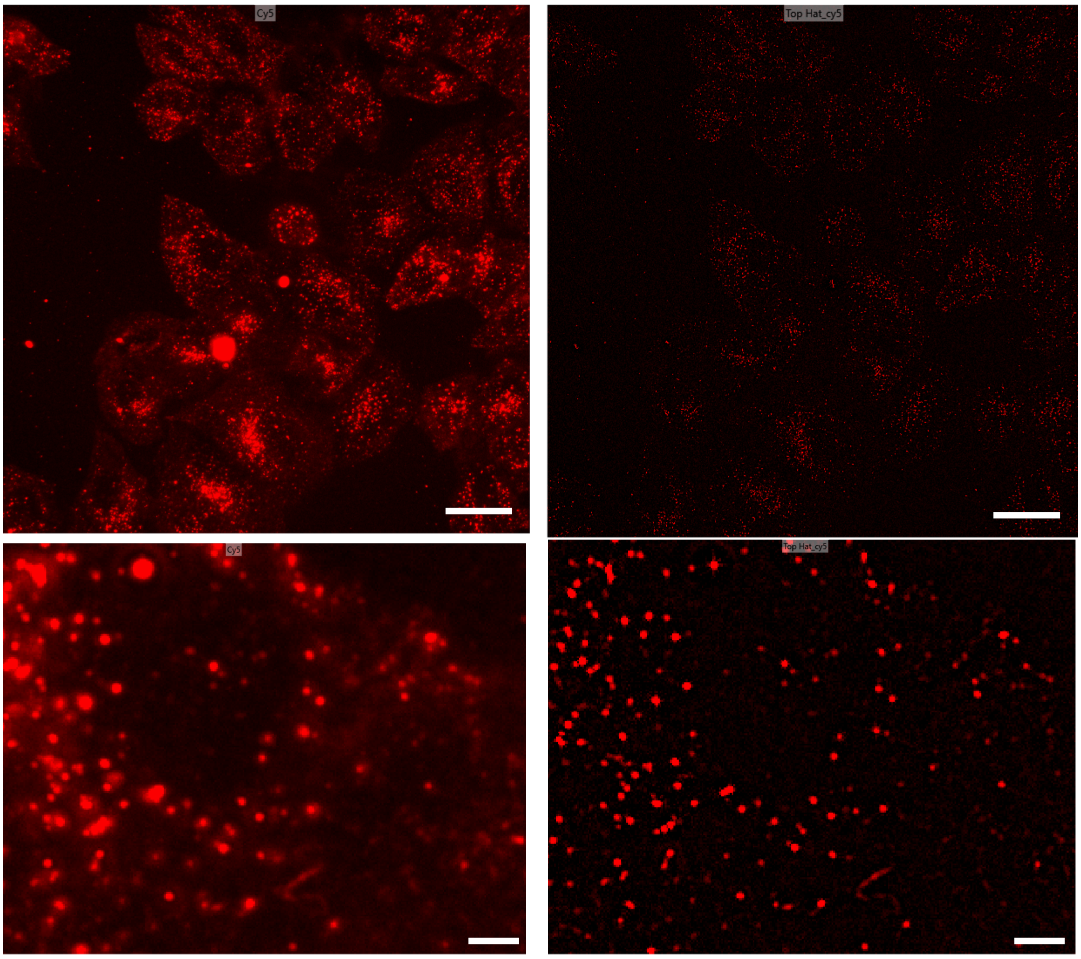

Figure S7. *Top-hat* transform (right) of the red channel image (left), scale bar = 30 µm. Bottom: zoomed in images, scale bar = 3.5 µm.

A mask based on the detected dots is applied to extract integrated and average intensity in the punctate structures, that most likely correspond to endosomes and lysosomes.

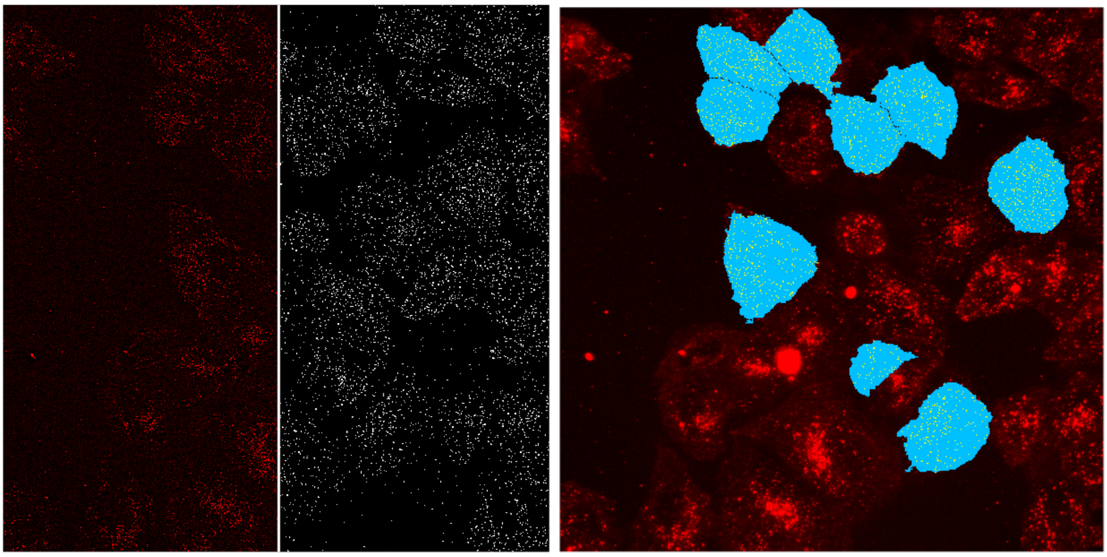

Figure S8. Segmentation of the dotted structures based on the *top-hat* transformation (left) and final mask applied on cells (right).

Data extracted from the "dots" mask are treated as described above for the cell mask.

4.3. Optimization of Uptake Conditions

Following the general procedure, HeLa Kyoto cells were incubated for 1, 2, 4 or 7 h with 500 nM or 1 μM **1** to determine optimal uptake conditions for inhibitor screening. Based on the images obtained, optimal conditions were deemed to be 2 h incubation with 500 nM **1**.

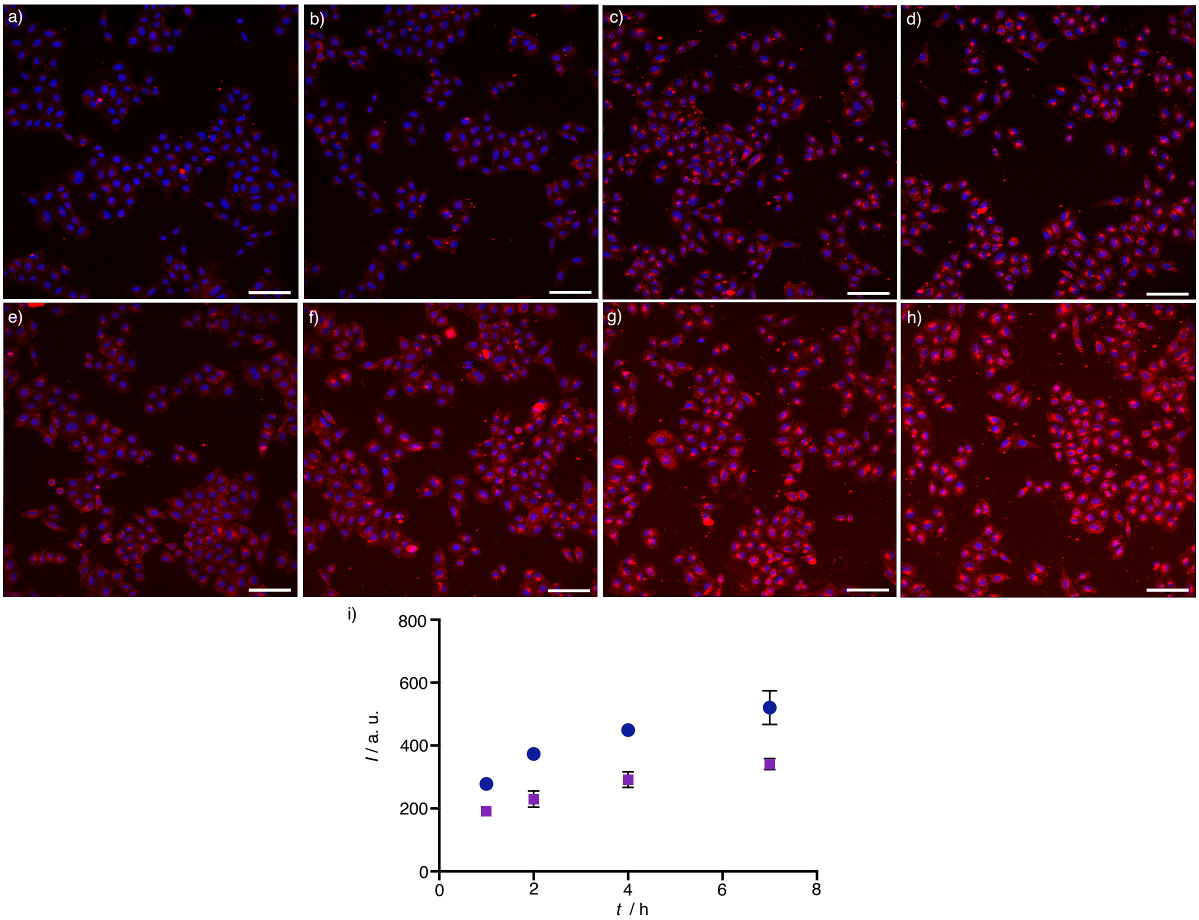

Figure S9. CLSM images of OPS **1** in HeLa Kyoto cells after 1, 2, 4 and 7 h incubation at 500 nM (a–d) or 1 µM (e–h) at 37 °C, scale bar = 100 µm. i) Automatically analyzed HCHT data showing normalized fluorescence intensity obtained from a) – h) with 500 nM (purple) or 1 µM (blue) **1**.

4.4. Inhibitor Screening

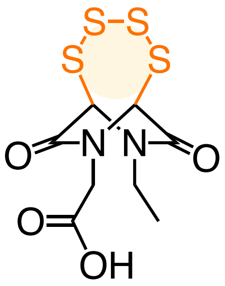

3

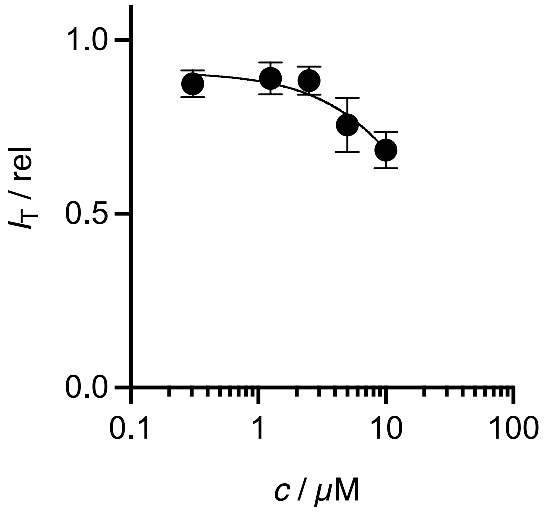

Figure S10. Automatically analyzed HCHT data showing relative fluorescence intensity ± SEM in HeLa Kyoto cells after pre-incubation with **3** for 1 h followed by washing and incubation with OPS **1** (1 μM) for 2 h.

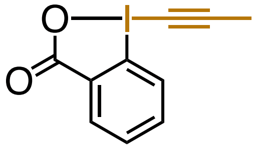

4

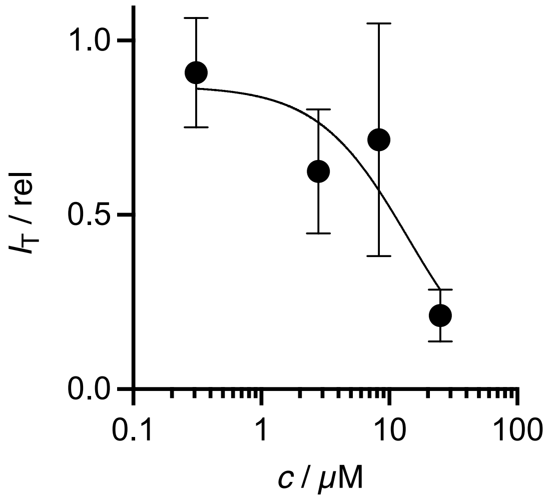

Figure S11. Automatically analyzed HCHT data showing relative fluorescence intensity ± SEM in HeLa Kyoto cells after pre-incubation with **4** for 1 h followed by washing and incubation with OPS **1** (500 nM) for 2 h.

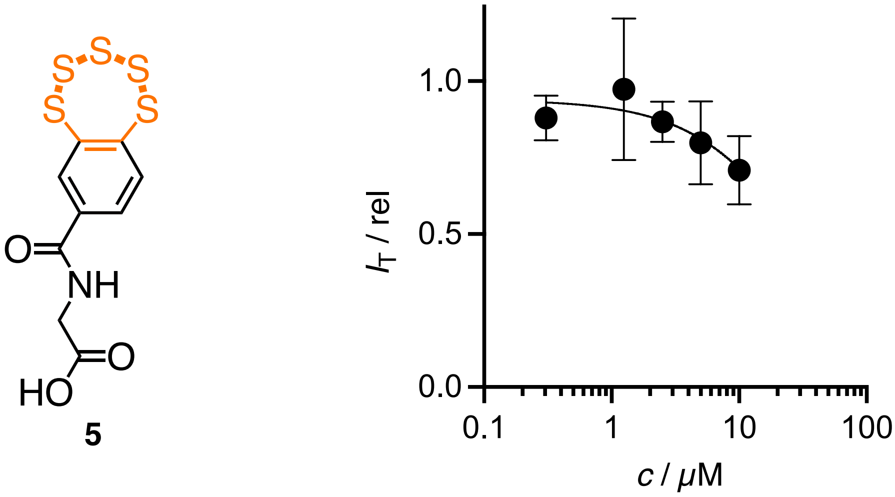

Figure S12. Automatically analyzed HCHT data showing relative fluorescence intensity ± SEM in HeLa Kyoto cells after pre-incubation with **5** for 1 h followed by washing and incubation with OPS **1** (1 μM) for 2 h.

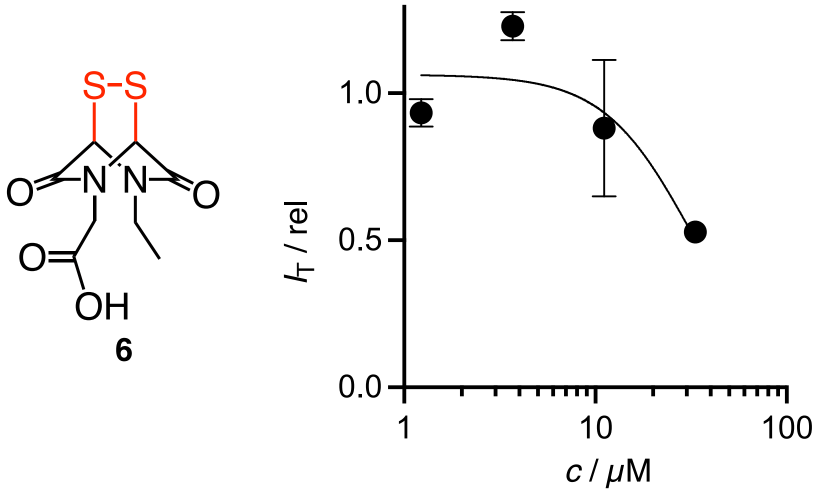

Figure S13. Automatically analyzed HCHT data showing relative fluorescence intensity ± SEM in HeLa Kyoto cells after pre-incubation with **6** for 1 h followed by washing and incubation with OPS **1** (500 nM) for 2 h.

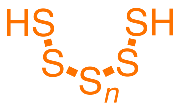

7

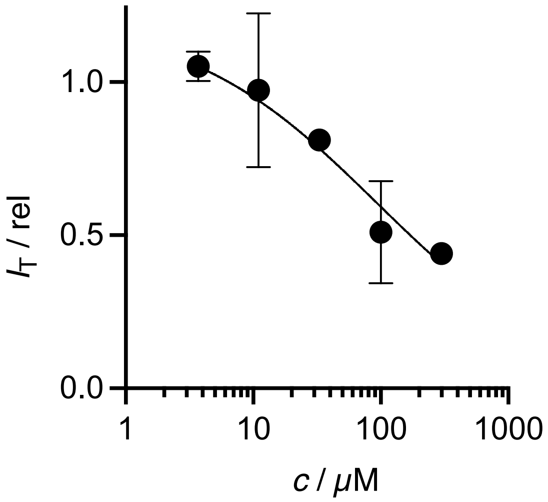

Figure S14. Automatically analyzed HCHT data showing relative fluorescence intensity ± SEM in HeLa Kyoto cells after pre-incubation with **7** for 1 h followed by washing and incubation with OPS **1** (500 nM) for 2 h.

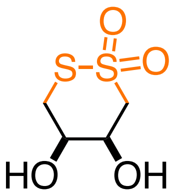

8

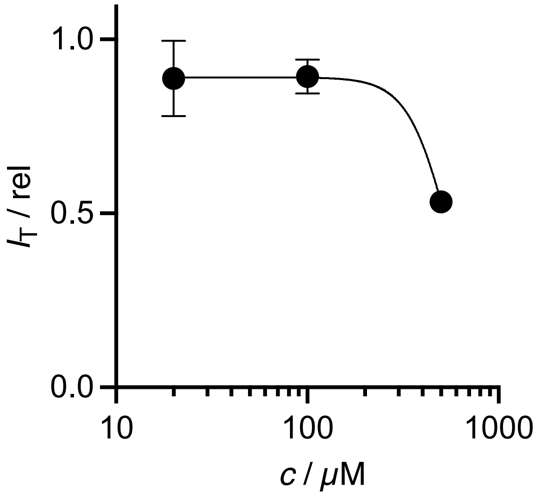

Figure S15. Automatically analyzed HCHT data showing relative fluorescence intensity ± SEM in HeLa Kyoto cells after pre-incubation with **8** for 1 h followed by washing and incubation with OPS **1** (500 nM) for 2 h.

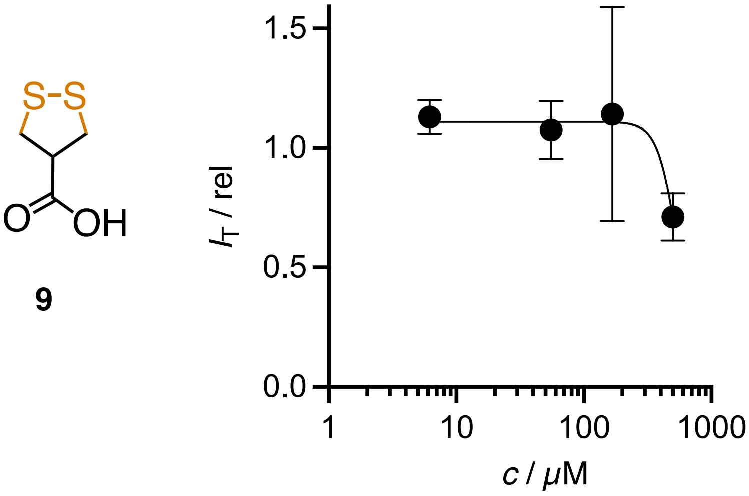

Figure S16. Automatically analyzed HCHT data showing relative fluorescence intensity ± SEM in HeLa Kyoto cells after pre-incubation with **9** for 1 h followed by washing and incubation with OPS **1** (500 nM) for 2 h.

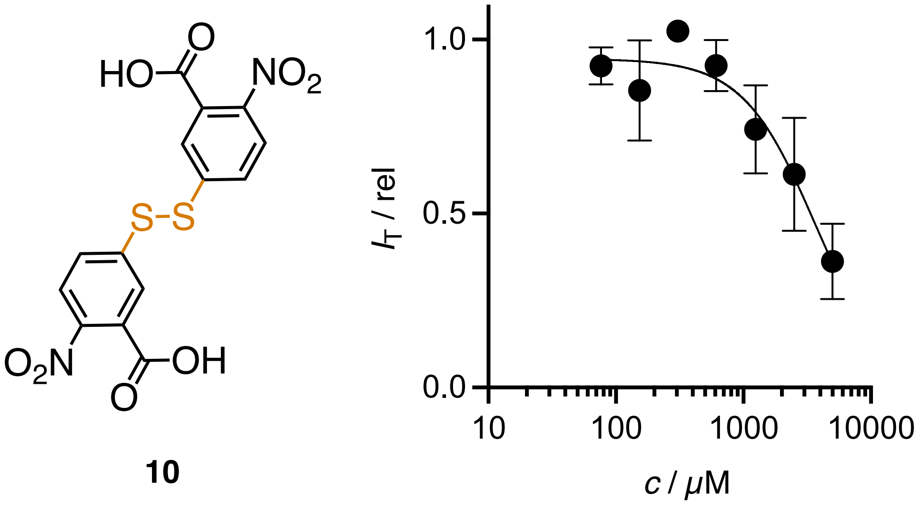

Figure S17. Automatically analyzed HCHT data showing relative fluorescence intensity ± SEM in HeLa Kyoto cells after pre-incubation with **10** for 1 h followed by washing and incubation with OPS **1** (250 nM) for 2 h.

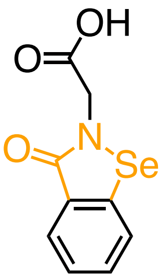

11

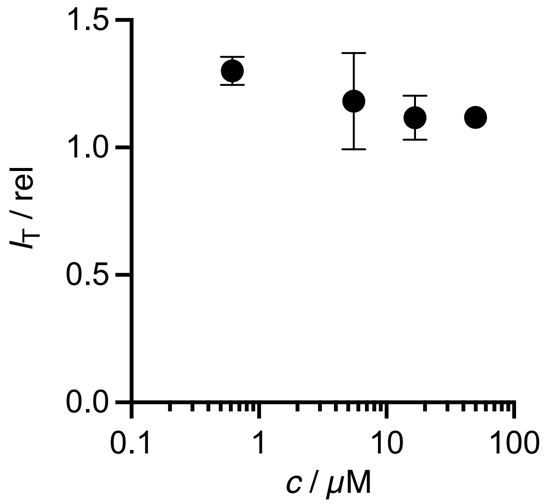

Figure S18. Automatically analyzed HCHT data showing relative fluorescence intensity ± SEM in HeLa Kyoto cells after pre-incubation with **11** for 1 h followed by washing and incubation with OPS **1** (500 nM) for 2 h.

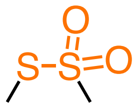

12

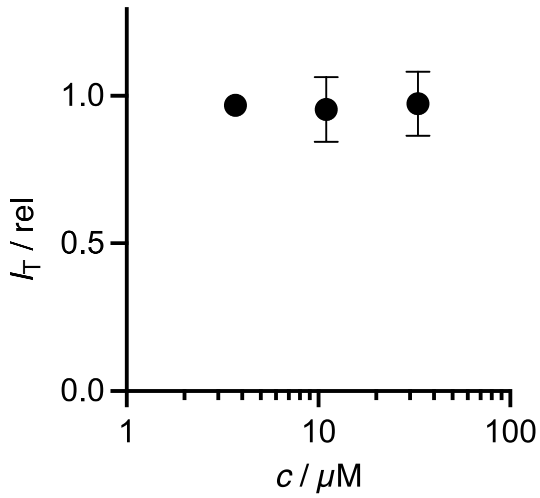

Figure S19. Automatically analyzed HCHT data showing relative fluorescence intensity ± SEM in HeLa Kyoto cells after pre-incubation with **12** for 1 h followed by washing and incubation with OPS **1** (250 nM) for 2 h.

Table S2. MIC and IC_{50} data of inhibitors **3-12**

Compound	c / nM[a]	MIC / µM[b]	IC_{50} / µM[c]	n[d]
**3**	1000	2	29	1.0
**4**	500	0.3	14	1.2
**5**	1000	3	32	1.0
**6**	500	15	31	1.9
**7**	500	24	165	0.6
**8**	500	250	550	4.0
**9**	500	450	550	5.4
**10**	250	870	3600	1.5
**11**	500	> 100	> 100	-
**12**	500	> 33	> 33	-

[a]Concentration of OPS **1**. [b]Minimum inhibitory concentration. [c]Half maximum inhibitory concentration. [d]Hill coefficient.

5. Activation of OPS Uptake

5.1. General Procedure for OPS Activation

Activation of OPS was performed by incubating **1** (500 nM) with the corresponding concentration of activator in PBS buffer pH 7.4 for 30 min at 25 °C prior to incubation.

When purification was performed to remove excess activator, the solution (500 µL) was concentrated down to 50 µL through Amicon Ultra 0.5 mL 3 kDa cutoff centrifugal filters, and diluted back to 500 µL with fresh PBS buffer. This process was repeated 7 times to remove excess activator and yield the solution of activated OPS (50 µL, 5 µM). Incubation with cells was then performed as described above.

5.2. HCHT Data of OPS Activation

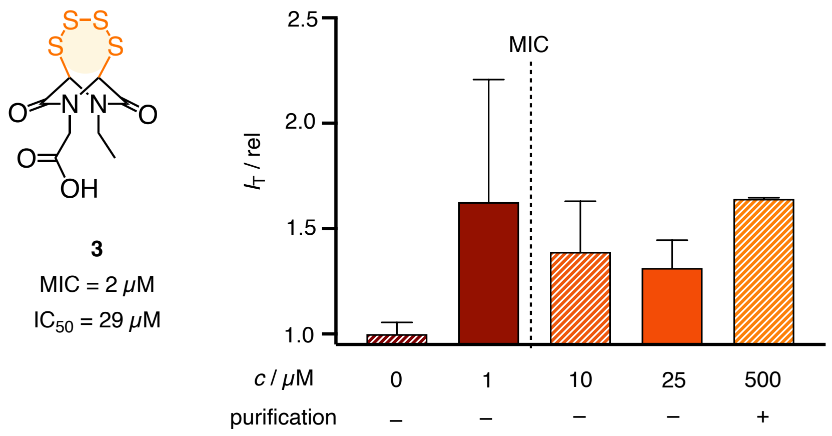

Figure S20. Automatically analyzed HCHT data showing relative fluorescence intensity ± SEM in HeLa Kyoto cells after incubation for 2 h with OPS **1** (500 nM) activated with 1, 10 or 25 µM **3** without purification, or 500 µM **3** followed by purification.

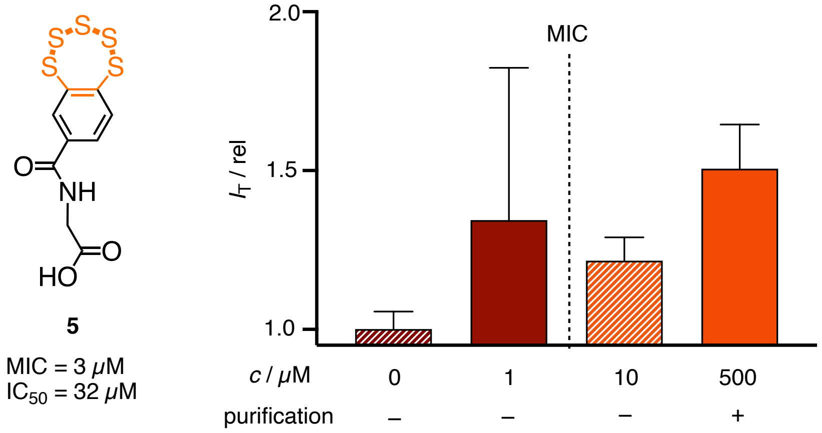

Figure S21. Automatically analyzed HCHT data showing relative fluorescence intensity in HeLa Kyoto cells after incubation for 2 h with OPS **1** (500 nM) activated with 1 or 10 µM **5** without purification, or 500 µM **5** followed by purification.

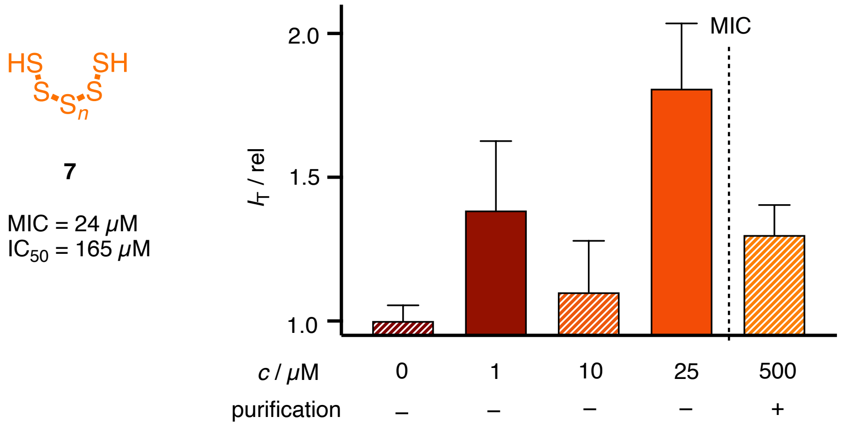

Figure S22. Automatically analyzed HCHT data showing relative fluorescence intensity in HeLa Kyoto cells after incubation for 2 h with OPS **1** (500 nM) activated with 1, 10 or 25 µM **7** without purification, or 500 µM **7** followed by purification.

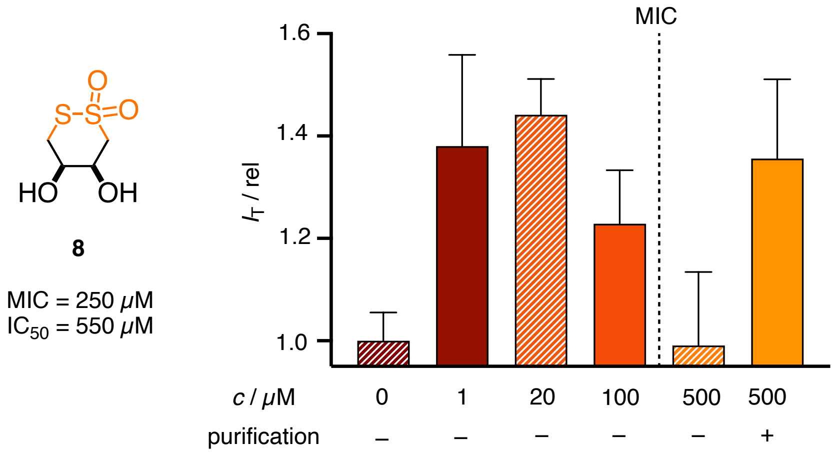

Figure S23. Automatically analyzed HCHT data showing relative fluorescence intensity in HeLa Kyoto cells after incubation for 2 h with OPS **1** (500 nM) activated with 1, 10, 20, 100 or 500 µM **8** without purification, or 500 µM **8** followed by purification.

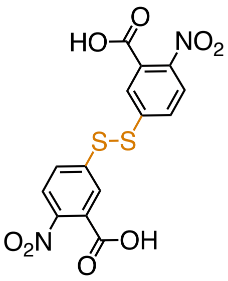

10

MIC = 870 μM
IC_{50} = 3.6 mM

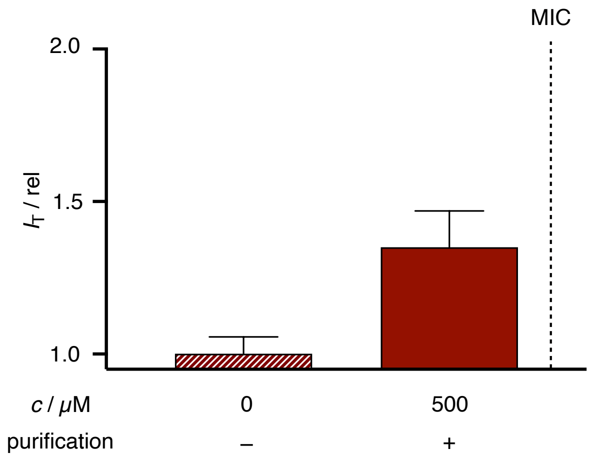

Figure S24. Automatically analyzed HCHT data showing relative fluorescence intensity in HeLa Kyoto cells after incubation for 2 h with OPS **1** (500 nM) activated with 500 µM **10** without purification.

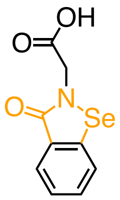

11

MIC > 100 μM

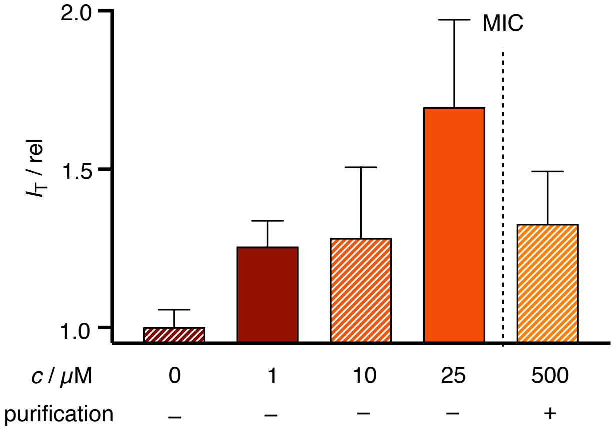

Figure S25. Automatically analyzed HCHT data showing relative fluorescence intensity in HeLa Kyoto cells after incubation for 2 h with OPS **1** (500 nM) activated with 1, 10 or 25 µM **11** without purification, or 500 µM **11** followed by purification.

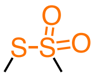

12

MIC > 33 μM

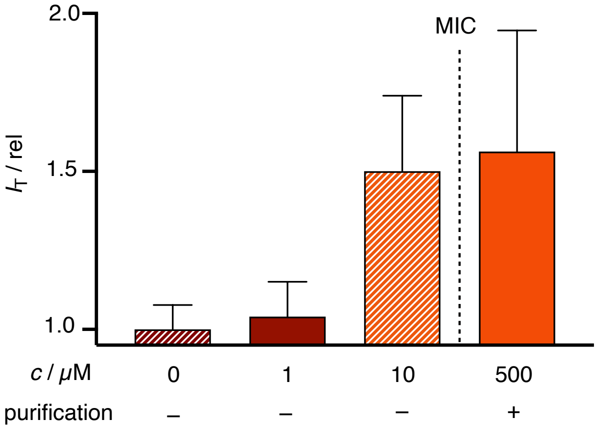

Figure S26. Automatically analyzed HCHT data showing relative fluorescence intensity in HeLa Kyoto cells after incubation for 2 h with OPS **1** (500 nM) activated with 1 or 10 µM **12** without purification or 500 µM **12** followed by purification.

6. HPLC Studies

6.1. General Procedure for HPLC Studies

Method A: Analytical HPLC were recorded using a JASCO LC-2000 Plus system equipped with quaternary pump (JASCO PU-2089), UV/Vis detector (JASCO UV-2077 Plus, λ_{abs} at 260 nm for detection) and fluorescence detector (JASCO FP-2020 Plus) equipped with a Jupiter 4u Proteo 90 Å 100 x 4.6 mm column, flow 0.5 mL/min with the following conditions: 95% H_2O/5% CH_3CN + 0.1% TFA for 2 min, then a linear elution gradient from 95% H_2O/5% CH_3CN + 0.1% TFA to 100% CH_3CN + 0.1% TFA in 8 min, 100% CH_3CN + 0.1% TFA for 3 min, then return to 95% H_2O/5% CH_3CN + 0.1% TFA.

Method B: Analytical HPLC were recorded using a JASCO LC-2000 Plus system equipped with quaternary pump (JASCO PU-2089), UV/Vis detector (JASCO UV-2077 Plus, λ_{abs} at 260 nm for detection) equipped with a Nucleosil 100-5 C_{18} AB column, flow 1.0 mL/min with the following conditions: linear elution gradient from 95% buffer/5% CH_3CN to 60% buffer/40% CH_3CN in 4 min, 60% buffer/40% CH_3CN for 1 min, then return to 95% buffer/5% CH_3CN. The buffer used was 100 mM TEAA, pH 7.5.

Method C: LCMS were recorded using a Thermo Scientific Accela HPLC equipped with a Thermo C18 Hypersil GOLD column (50 x 2.1 mm, 1.9 µm particles size) coupled with a LCQ Fleet three-dimensional ion trap mass spectrometer (ESI, Thermo Scientific) with a linear elution gradient from 95% H_2O / 5% CH_3CN + 0.1% TFA to 10% H_2O / 90% CH_3CN + 0.1% TFA in 4.0 min at a flow rate of 0.75 mL/min. UV (λ_{abs} = 260 nm) detection was used.

6.2. Activation of BPS Library

Compounds 18. To a solution of **5a** in DMSO (100 µM, 4 µL, 1.0 eq.) was added a solution of **17** in PBS buffer (5.56 µM (0.5 eq.), 11.1 µM (1.0 eq.), 22.2 µM (2.0 eq.), 111 µM (10 eq.), or 1.11 mM (100 eq.), 36 µL, pH 7.4). HPLC chromatograms were measured after 30 min at rt using method A.

6.3. Di- and Polysulfide Exchange with 5'-AMPS

Compound 22. To a solution of 5'-AMPS **13** in PBS (1 mM, 4.00 µL, 1.0 eq.) was added a solution of **3** in PBS buffer (111 µM (1 eq.), 1.11 mM (10 eq.), 11.1 mM (100 eq.), 36 µL, pH 7.4). HPLC chromatograms were measured after 30 min using method B.

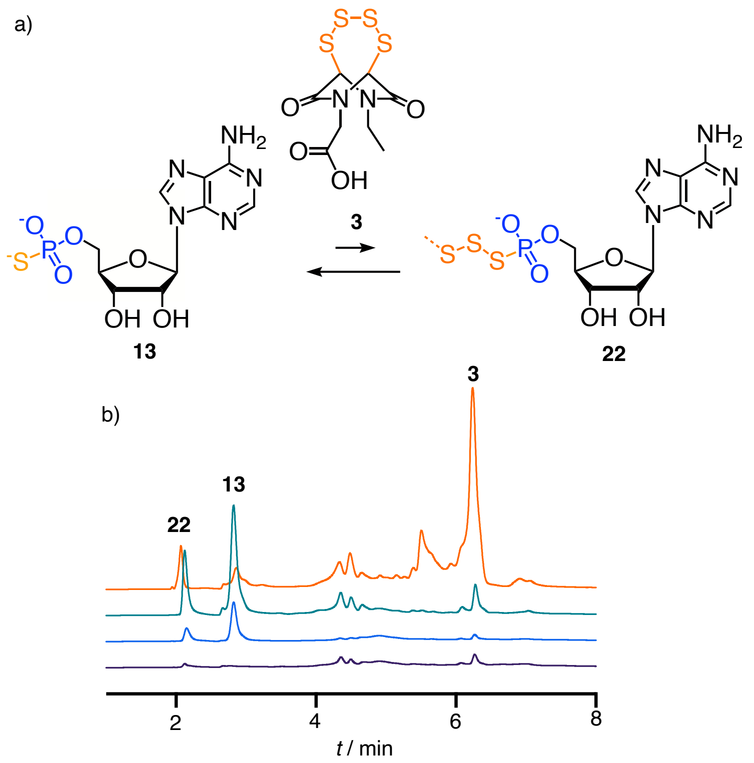

Figure S27. a) Reaction of 5'-AMPS **13** with **3**. b) HPLC chromatograms of ETP-S4 **3** (purple) and **13** with 1.0 (blue), 10 (green) and 100 (orange) equiv. of **3**.

Compound 23. To a solution of 5'-AMPS **13** in PBS (1 mM, 4.00 µL, 1.0 eq.) was added a solution of **7** in PBS buffer (111 µM (1 eq.), 1.11 mM (10 eq.), 11.1 mM (100 eq.), 111 mM (1000 equiv.), 36 µL, pH 7.4). HPLC chromatograms were measured after 30 min using method B.

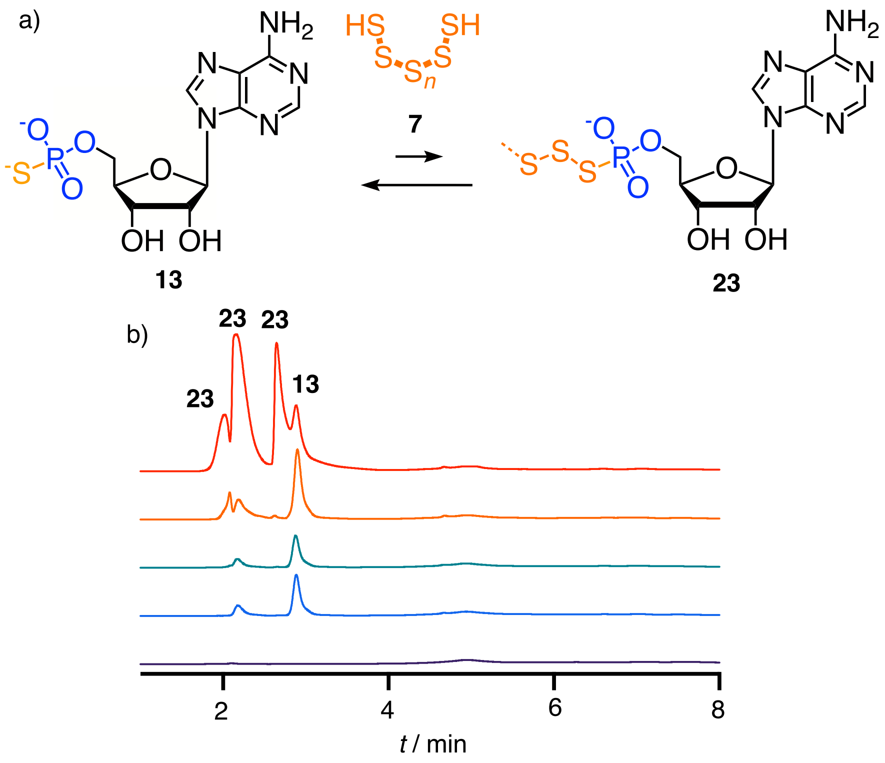

Figure S28. a) Reaction of 5'-AMPS **13** with **7**. b) Normalized HPLC chromatograms of **7** (purple) and **13** with 1.0 (blue), 10 (green), 100 (orange) and 1000 (red) equiv. of **7**.

Compound 24. To a solution of 5'-AMPS **13** in PBS (1 mM, 4.00 µL, 1.0 eq.) was added a solution of DTNB **10** in PBS buffer (11.1 µM (0.1 eq.), 111 µM (1 eq.), 1.11 mM (10 eq.), 11.1 mM (100 eq.), 36 µL, pH 7.4). HPLC chromatograms were measured after 30 min using method A. HRMS (ESI, +ve) calcd for $C_{17}H_{17}N_6O_{10}PS_2$ $[M+2H]^+$: 561.0259, found: 561.0275.

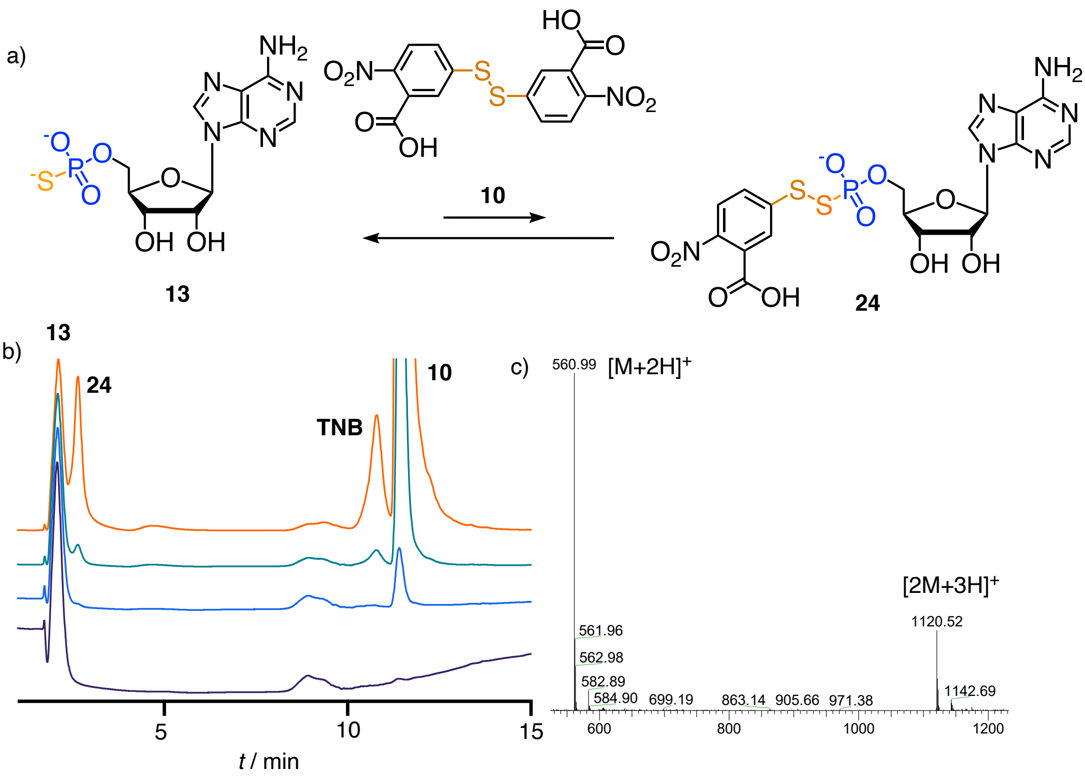

Figure S29. a) Reaction of 5'-AMPS **13** with DTNB **10**. b) Normalized HPLC chromatograms of **13** with 0.1 (purple), 1.0 (blue), 10 (green) and 100 (orange) equiv. of **10**. c) ESI-MS spectrum of exchange product **24**.

Compound 16. To a solution of 5'-AMPS **13** in PBS (1 mM, 4.00 µL, 1.0 eq.) was added a solution of thiosulfonate **12** in PBS buffer (11.1 µM (0.1 eq.), 22.2 µM (0.2 eq.), 55.5 µM (0.5 eq.), 111 µM (1 eq.), 444 µM (4 eq.) or 1.11 mM (10 eq.), 36 µL, pH 7.4). HPLC chromatograms were measured after 30 min using method C. HRMS (ESI, +ve) calcd for $C_{11}H_{16}N_5O_6PS_2$ $[M+2H]^+$: 410.0353, found: 410.0367.

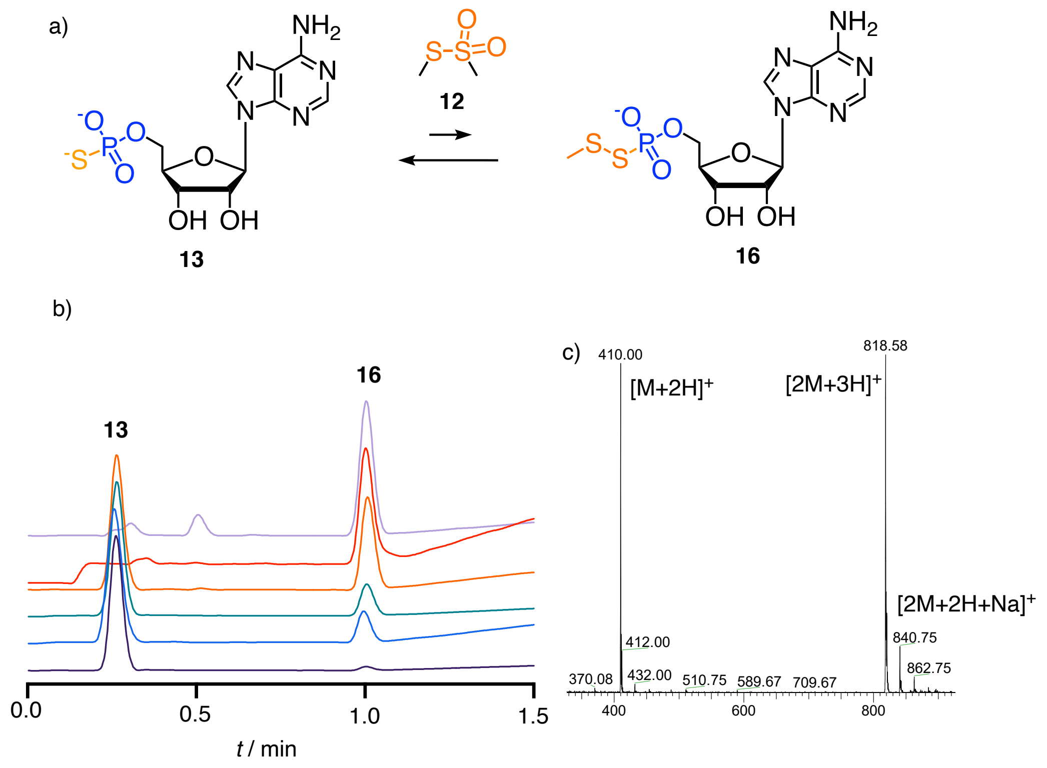

Figure S30. a) Reaction of 5'-AMPS **13** with thiosulfonate **12**. b) Normalized HPLC chromatograms of **13** with 0.1 (purple), 0.2 (blue), 0.5 (green), 1.0 (orange), 4.0 (red) and 10 (lavender) equiv. of **12**. c) ESI-MS spectrum of exchange product **16**.

Compound 15b. To a solution of 5'-AMPS **13** in PBS (1 mM, 4.00 µL, 1.0 eq.) was added a solution of cystine **14b** in PBS buffer (111 µM (1 eq.), 1.11 mM (10 eq.), 11.1 mM (100 eq.), 111 mM (1000 equiv.), 36 µL, pH 7.4). HPLC chromatograms were measured after 30 min using method B. HRMS (ESI, -ve) calcd for $C_{19}H_{29}N_6O_{10}PS_2$ $[M]^-$: 595.1051, found: 595.1029.

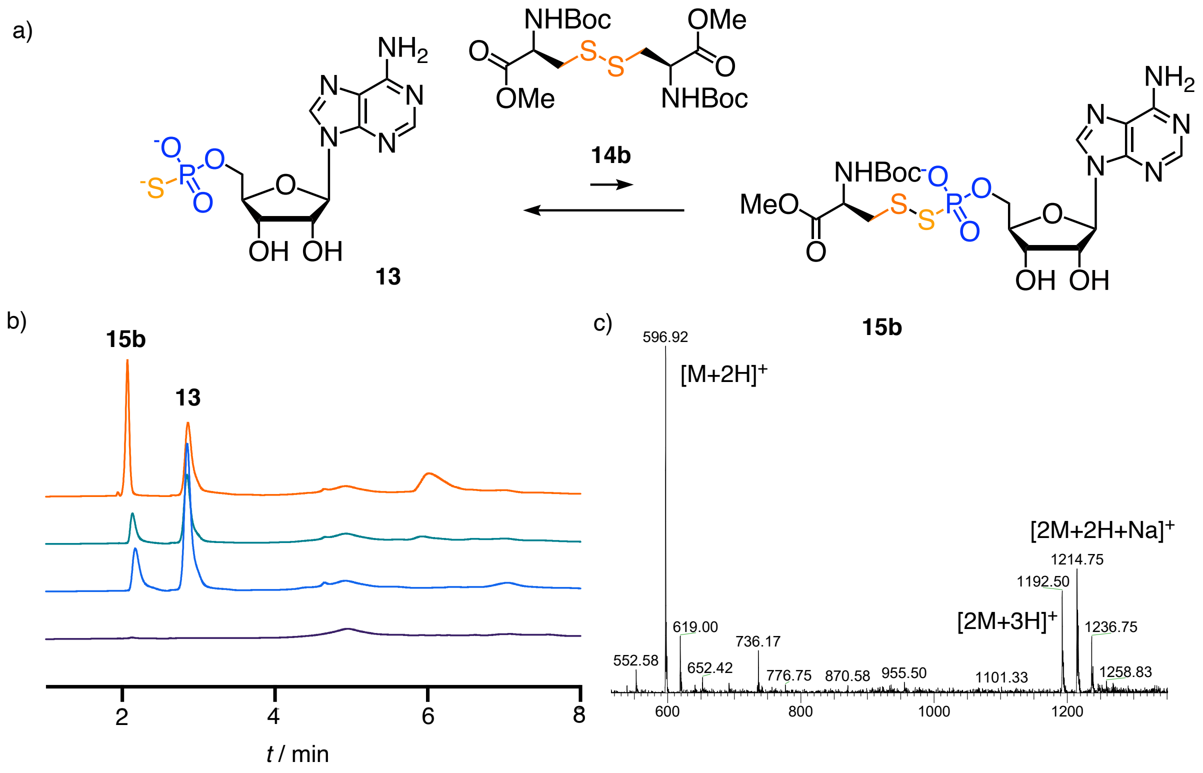

Figure S31. a) Reaction of 5'-AMPS **12** with cystine **14b**. b) HPLC chromatograms of **14b** (purple) and **12** with 1 (blue), 10 (green) and 100 (orange) equiv. of **14b**. c) ESI-MS spectrum of exchange product **15b**.

Compound 15a. To a solution of cystine **14a** in PBS (1 mM, 4.00 µL, 1.0 eq.) was added a solution of 5'-AMPS **13** in PBS buffer (111 µM (1 eq.), 1.11 mM (10 eq.), 11.1 mM (100 eq.), 36 µL, pH 7.4). HPLC chromatograms were measured after 30 min using method C. HRMS (ESI, +ve) calcd for $C_{30}H_{30}N_7O_7PS_2$ $[M+2H]^+$: 696.1459, found: 696.1446.

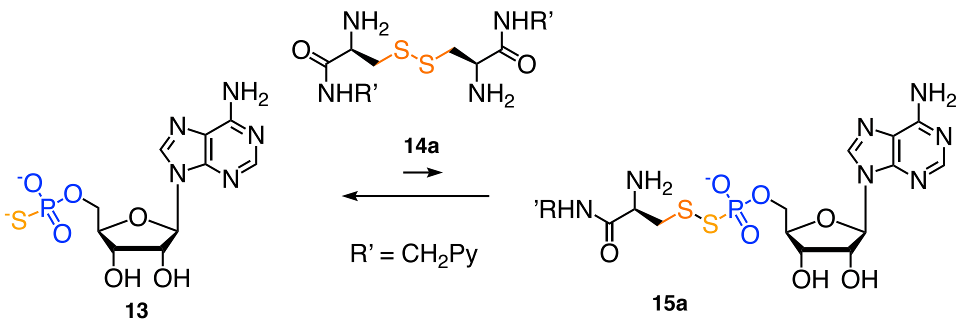

Scheme S2. Reaction of 5'-AMPS **13** with cystine **14a**.

Cysteine **25** was generated *in situ* by mixing cystine **14a** with 1 equivalent of TCEP. To a solution of cysteine **25** in PBS (1 mM, 4.00 µL, 1.0 eq.) was added a solution of 5'-AMPS **13** in PBS buffer (111 µM (1 eq.), 1.11 mM (10 eq.), 11.1 mM (100 eq.), 36 µL, pH 7.4). HPLC chromatograms were measured after 30 min using method C.

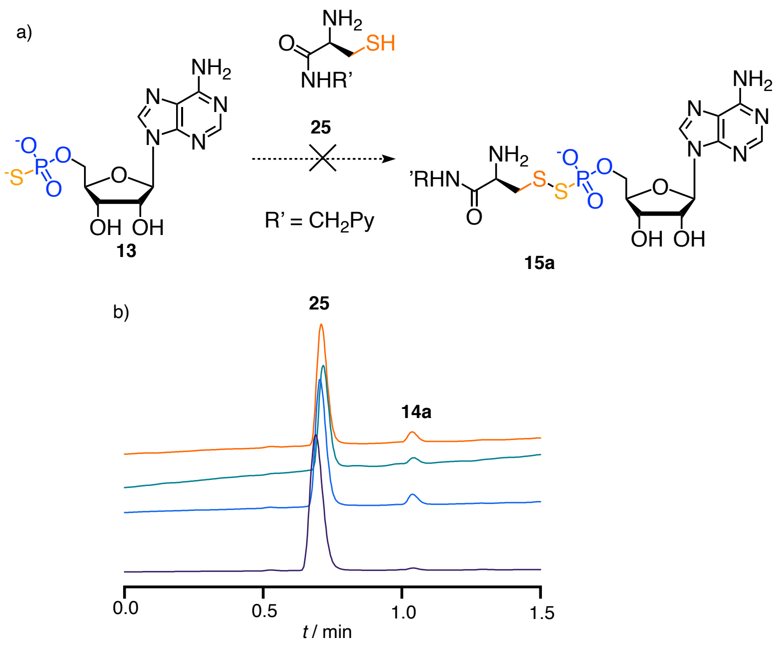

Figure S32. a) Reaction of 5'-AMPS **13** with cysteine **25**. b) HPLC chromatograms of **25** (purple) and **25** with 1 (blue), 10 (green) and 100 (orange) equiv. of **13**.

7. NMR Spectra

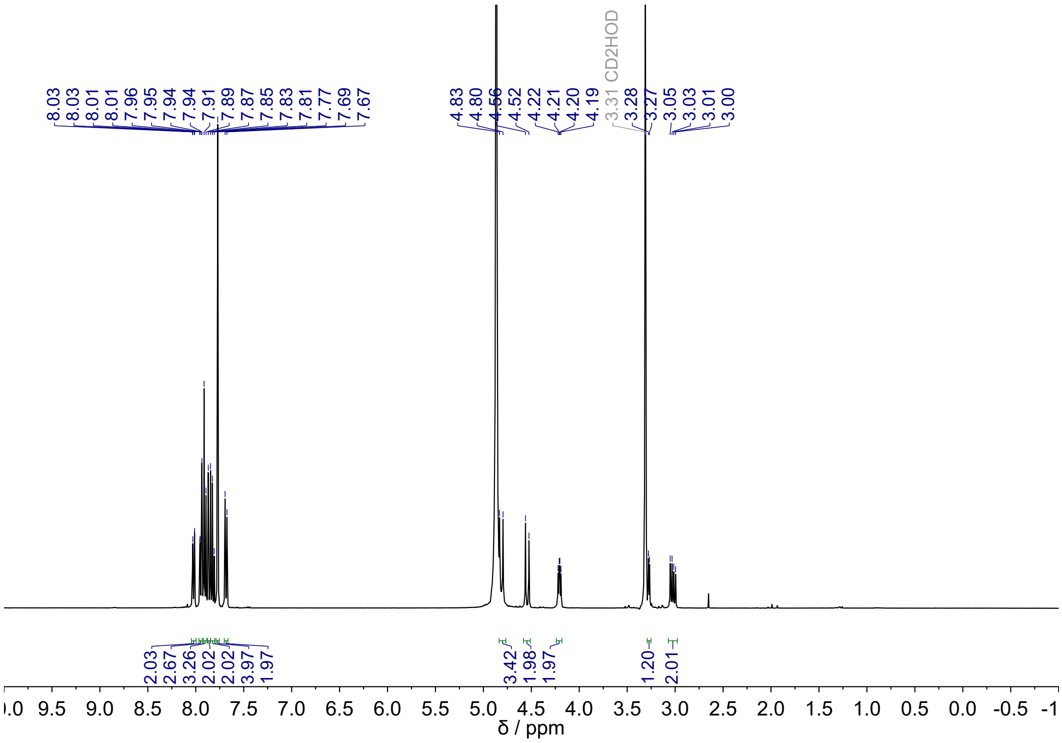

Figure S33. 400 MHz ^{1}H NMR spectrum of **14a** in CD_3OD.

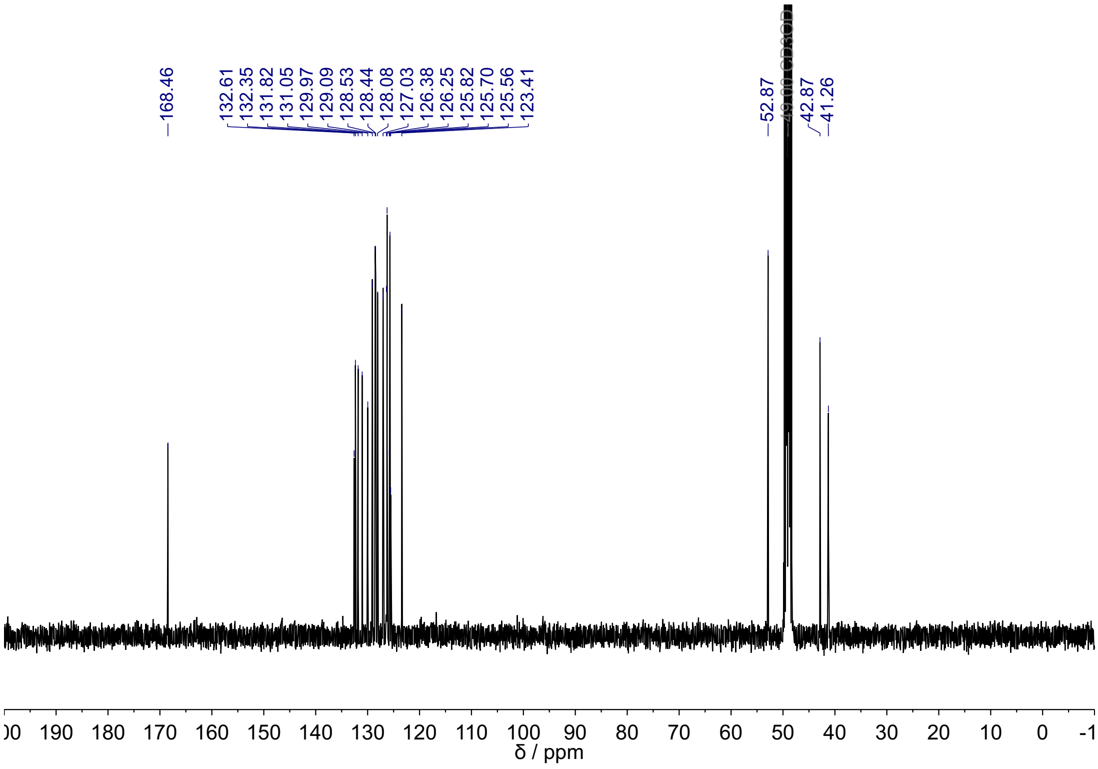

Figure S34. 101 MHz ^{13}C NMR spectrum of **14a** in CD_3OD.

8. Supporting References

[S1] Y. Cheng, A.-T. Pham, T. Kato, B. Lim, D. Moreau, J. López-Andarias, L. Zong, N. Sakai, S. Matile, *Chem. Sci.* **2021**, *12*, 626–631.

[S2] R. Frei, M. D. Wodrich, D. P. Hari, P.-A. Borin, C. Chauvier, J. Waser, *J. Am. Chem. Soc.* **2014**, *136*, 16563–16573.

[S3] W. B. Jin, C. Xu, Q. Cheng, X. L. Qi, W. Gao, Z. Zheng, E. W. C. Chan, Y.-C. Leung, T. H. Chan, K.-Y. Wong, S. Chen, K.-F. Chan, *Eur. J. Med. Chem.* **2018**, *155*, 285–302.

The original data can be found at: https://dx.doi.org/10.5281/zenodo.5018160
